# OptMAVEn-2.0: De novo Design of Variable Antibody Regions against Targeted Antigen Epitopes

**DOI:** 10.3390/antib7030023

**Published:** 2018-06-30

**Authors:** Ratul Chowdhury, Matthew F. Allan, Costas D. Maranas

**Affiliations:** 1 Department of Chemical Engineering, The Pennsylvania State University, State College, PA 16802, USA; ratul@psu.edu (R.C.); mfallan@mit.edu (M.F.A.); 2 Computational and Systems Biology Initiative, Massachusetts Institute of Technology, Cambridge, MA 02139, USA

**Keywords:** de novo antibody design, zika envelope protein, computational protein design, specific antigen epitope

## Abstract

Monoclonal antibodies are becoming increasingly important therapeutic agents for the treatment of cancers, infectious diseases, and autoimmune disorders. However, laboratory-based methods of developing therapeutic monoclonal antibodies (e.g., immunized mice, hybridomas, and phage display) are time-consuming and are often unable to target a specific antigen epitope or reach (sub)nanomolar levels of affinity. To this end, we developed Optimal Method for Antibody Variable region Engineering (OptMAVEn) for de novo design of humanized monoclonal antibody variable regions targeting a specific antigen epitope. In this work, we introduce OptMAVEn-2.0, which improves upon OptMAVEn by (1) reducing computational resource requirements without compromising design quality; (2) clustering the designs to better identify high-affinity antibodies; and (3) eliminating intra-antibody steric clashes using an updated set of clashing parts from the Modular Antibody Parts (MAPs) database. Benchmarking on a set of 10 antigens revealed that OptMAVEn-2.0 uses an average of 74% less CPU time and 84% less disk storage relative to OptMAVEn. Testing on 54 additional antigens revealed that computational resource requirements of OptMAVEn-2.0 scale only sub-linearly with respect to antigen size. OptMAVEn-2.0 was used to design and rank variable antibody fragments targeting five epitopes of Zika envelope protein and three of hen egg white lysozyme. Among the top five ranked designs for each epitope, recovery of native residue identities is typically 45–65%. MD simulations of two designs targeting Zika suggest that at least one would bind with high affinity. OptMAVEn-2.0 can be downloaded from our GitHub repository and webpage as (links in Summary and Discussion section).

## 1. Introduction

Antibodies are versatile molecules produced in B-cells and have become the basis of many therapeutics [1,2,3] and diagnostics [4,5,6] for cancers [6,7,8], infectious diseases [9], and autoimmune disorders [10]. They are affinity proteins that are crucial for humoral immunity and are able to bind to foreign proteins with high specificity [11]. Administration of serum from survivors to treat patients during infectious disease outbreaks such as the 1918 influenza pandemic [12] marks the early years of antibody-mediated therapeutics. The first monoclonal antibodies were developed by immunizing mice with a target antigen [6]. However, high immunogenicities of murine antibodies limit their efficacies in humans [6]. Subsequent efforts have resulted in chimeric constructs [6] of murine variable domains grafted onto human constant domains. Although chimeras exhibit less immunogenicity relative to fully murine antibodies [6], they are not entirely human [6] and may still cause adverse reactions. Methods such as phage display [13] and yeast display [14] have been able to create high-affinity, completely humanized antibodies. However, all experimental methods antibody development are time-consuming [15], and none offers a general approach to target a specific antigen epitope, increase affinity without increasing immunogenicity, and categorize designs based on the primary sequence of the variable domain and the binding pose of the antigen [16].

Computational methods of antibody design have addressed these limitations. Software exists for designing stable antibody-antigen complexes [17,18,19], predicting the immunogenicities of antibody sequences [20,21], and predicting stabilizing mutations to the antibody complimentary determining regions (CDRs) [17,22,23,24]. Before our work, we knew of no software that could design antibodies de novo—that is, without an initial structure of an antibody bound to the antigen [17,18,19]. To this end, we first developed OptCDR [17], which designed de novo CDRs of high affinities but not low immunogenicities. This limitation was addressed in the following effort, OptMAVEn [16], which designs full antibody variable domains. Two subsequent efforts at antibody design were AbDesign [18] by Lapidoth et al. and Rosetta Antibody Design (RAbD) [19] by Adolf-Bryfogle et al. However, both of these tools build upon existing antibodies and thus require an initial structure of the antigen-antibody complex.

In addition to designing antibodies without an input structure, OptMAVEn-2.0 performs computational affinity maturation while avoiding sequences likely to trigger an immune response. During affinity maturation, OptMAVEn mimics natural mutation preferences by mutating residues in the CDRs with three times the frequency compared to residues in the framework regions. OptMAVEn screens a large set of antigen poses, designs antibodies for each pose, and outputs the designs with the most favorable antigen-antibody interaction energies. However, OptMAVEn’s large computational time and storage requirements limit sampling of antigen poses, which reduces the likelihood of finding designs with favorable interaction energies.

Here, we introduce OptMAVEn-2.0, which is capable of sampling a larger set of antigen poses within roughly one day, while OptMAVEn required over one week. Each antibody variable region comprises a heavy (H) and a light (L, or kappa-K) chain. An end to end joined variable (V), a complimentarity determining region (CDR3), and a joining (J) region constitutes each heavy and light chain. We have retained the mixed-integer linear programming (MILP) core module, which identifies six optimal parts from the Modular Antibody Parts (MAPs) database [25] (HV, HCDR3, HJ, L/KV, L/KCDR3, and L/KJ) that constitute the variable domain. While OptMAVEn requires excessive disk storage by storing each antigen pose as a separate Protein Data Bank (PDB) file, OptMAVEn-2.0 alleviates this problem by storing only one reference pose and using transformation matrices to generate other poses as needed.

OptMAVEn-2.0 introduces a systematic procedure to classify antibody designs. Each MAPs part is assigned a three-dimensional coordinate that depends on the sequence similarity to other MAPs parts of the same type (HV, HCDR3, and so on). We compute a matrix of pairwise sequence similarity scores for each type of MAPs parts and then convert similarities into metric distances using Stojmirovic’s method [26]. We use Distance Geometry Optimization Software (DGSOL 1.3, Argonne National Laboratory, Lemont, IL, USA) [27] to embed these distances in 3D-Euclidean space, yielding a 3D-coordinate for each MAPs part [28]. After relaxing all designs, OptMAVEn-2.0 creates for each design a 23-dimensional vector consisting of the 3D-coordinates of its six MAPs parts (18 dimensions), the epitope centroid (three dimensions), and the sine and cosine of the antigen z angle (two dimensions). A Principal Component Analysis (PCA) step transforms these 23-dimensional vectors into three-dimensional vectors, which are then used in *k*-means clustering of the designs. OptMAVEn-2.0 then ranks the designs from most to least promising by cycling through the clusters and selecting the top design from each cluster until all designs have been selected.

After ranking these germline designs (so named because they are assembled from MAPs parts that correspond to germline genes), the user has the option of assessing the stability of the germline designs bound to the epitope of interest using short (25 ns) molecular dynamics (MD) trajectories (using QwikMD [29]) and/or subjecting the designs for in silico affinity maturation while ensuring that the immunogenicity scores are reduced. The MD step assesses the stability of the most promising designs over 50 ns, ensuring that the best antibody designs bind stably to the antigen. Affinity maturation is implemented within Iterative Protein Redesign and Optimization (IPRO) software [30] and optimizes affinities of germline designs while ensuring that their immunogenicity does not increase. The immunogenicity of each design is assessed using the “human string content” (HSC) [20], which estimates the potential of a sequence to elicit a T cell response when presented on Major Histocompatibility Complex (MHC)-II. HSC is used to calculate a “humanization score” (HScore) [16]: an antibody with a low HScore is relatively humanized and thus has low potential to trigger an immune response in the human body.

We used OptMAVEn-2.0 to design antibodies targeting five epitopes of Zika envelope (E) protein and three of hen egg white lysozyme. We assessed the stability of two designs from one of the Zika cases using short MD simulations. Recovery of epitope-binding residues and sequence similarities are reported for the top five designs for all the other cases.

## 2. Methods

### 2.1. Overview

**Figure 1:**
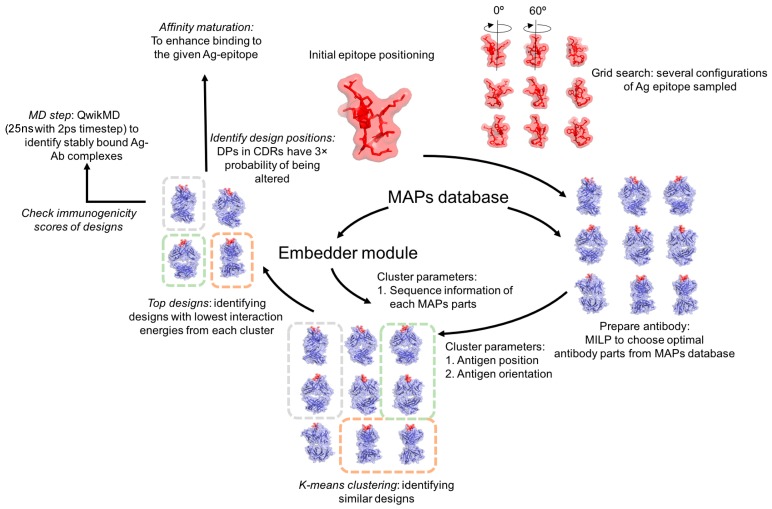
The workflow of OptMAVEn-2.0. First, the initial epitope positioning step rotates the antigen such that its epitope points downward with epitope centroid at the origin. The grid search step generates an ensemble of antigen positions, followed by the mixed-integer linear programming (MILP) step, where the six lowest interaction energy Modular Antibody Parts (MAPs) are chosen to construct the variable antibody fragment. A Euclidean coordinate for each part in the MAPs database was generated using the embedder module. The *k*-means protocol uses these and the epitope centroid coordinates and rotation angle to cluster the antibodies. The antibodies with the most negative MILP energy in each cluster are then subjected to structural relaxation and a short molecular dynamics (MD) routine to verify their high affinities. Stable designs emerging from this step could be affinity matured with the dual objective of enhancing their antigen-antibody affinities and lowering their immunogenic potentials.

OptMAVEn-2.0 (Figure 1) is de novo antibody design software that extends OptMAVEn [16]. OptMAVEn-2.0 is fully automated (unlike OptMAVEn), requires less CPU time and disk storage, and features a novel clustering algorithm to increase the diversity of antibody designs raised against a specific antigen epitope. Both versions assemble antibodies from the MAPs database of antibody parts [25], which contains variable (V), CDR3, and joining (J) regions for the heavy (H), lambda (L), and kappa (K) chains. First, the user specifies the antigen and its epitope. As in OptMAVEn, the antigen is rotated such that its epitope faces a framework antibody, and then an ensemble of antigen positions is generated by translating and rotating the antigen within a user-defined antigen binding site. Positions in which the antigen clashes with the framework antibody are discarded. At each remaining antigen position, the interaction energy between the antigen and each part in the MAPs database is calculated, and a set of six non-clashing MAPs parts is selected so as to minimize the sum of the interaction energies between the parts and the antigen. These associations of an antigen position with a set of MAPs parts (i.e., designs) are clustered using a *k*-means approach. OptMAVEn-2.0 sequentially scans through all clusters, generating a Protein Data Bank (PDB) and FASTA file of the design with the most negative interaction energy in each cluster, repeating until files have been created for all designs. These designs can then undergo further validation (e.g., QwikMD [29]) or sequence optimization (e.g., affinity maturation and reduction of HScore [16]) to yield a set of designs for experimental validation or optimization (e.g., with phage display [13]).

### 2.2. Design and Implementation

OptMAVEn-2.0 runs continuously from the initial step (starting an experiment) to the output of germline designs. This feature reduces the effort on the part of the user and also makes OptMAVEn-2.0 easier to use than OptMAVEn, which required manual initiation of each step in the workflow. OptMAVEn-2.0 is currently supported on UNIX platforms with Python 2.7 [31], NumPy [32], SciPy [33], and BioPython 1.7 [34]. Within its main directory, OptMAVEn-2.0/, are subdirectories src/(source modules written in Python and Tool Command Language (TCL) scripts), experiments/(all experiment directories), and data/(files of antigen structures, topologies, and parameters). If the directory experiments/ does not exist, it is created automatically when the first experiment is started. The data/directory contains three subdirectories: (1) pdbs/stores structures of antigens, which may be in either PDB or mmCIF format; (2) input_files/stores topology and parameter files needed for energy calculations in CHARMM (Chemistry at Harvard Molecular Mechanics) [35]; and (3) antibodies/stores framework antibody structures and the MAPs database. Before an experiment can be started, the structure of the antigen and all required topology and parameter files must be located in pdbs/and input_files/, respectively. OptMAVEn-2.0 is pre-installed with default CHARMM topology (top_all27_prot_na.rtf) and parameter (par_all27_prot_na.prm) files. The user may add additional files to support a wider range of antigens (or small drug molecules) that characterize these molecules’ types of bonds, angles, dihedrals and improper dihedral angles. An *./OptMAVEn-2.0* executable is also present in the OptMAVEn-2.0/main directory and is used to initiate an experiment.

### 2.3. Starting an Experiment

To start an experiment, the user enters *./OptMAVEn-2.0* into a UNIX terminal from the main directory of OptMAVEn-2.0. First, the user names the experiment. OptMAVEn-2.0 creates a directory named OptMAVEn-2.0/experiments/name to hold all of the experiment’s results and temporary files. The user may customize the configuration of the experiment (e.g., by specifying topology and parameter files) or use the default configuration, defined in OptMAVEn-2.0/src/standards.py. The user then specifies the file containing the antigen’s structure, the chains that constitute the antigen, heteroatoms to exclude, and the residues of each chain that constitute the epitope region for which the antibody is to be designed. For each antigen chain, at least one epitope residue must be selected.

OptMAVEn-2.0 preprocesses the user-specified antigen structure file by automatically removing heteroatoms and chains that are not part of the antigen but are present in the crystal structure obtained from the Protein Data Bank (PDB). This feature makes initiating an experiment simpler. Unlike OptMAVEn-2.0, in the older OptMAVEn, the user must remove these chains and heteroatoms manually and create a file listing the epitope residues; OptMAVEn does not check that these residues actually exist, but OptMAVEn-2.0 does. In OptMAVEn-2.0, users select antigen chains, heteroatoms, and epitope residues using a simple, single-line syntax. Ranges are indicated with hyphens, while individual items are delimited with commas: for example, A–C, E specifies chains A, B, C, and E of a certain molecule. Furthermore, OptMAVEn-2.0 makes it simpler for the user by listing the available chains of the antigen molecule to choose from. Overall, unlike in OptMAVEn, the user needs to know only the antigen PDB accession ID and the residues that constitute the epitope of interest. OptMAVEn-2.0 automatically downloads the molecule from the Protein Data Bank using a package in BioPython [34] and then performs the remaining steps.

### 2.4. Antigen Positioning

OptMAVEn-2.0 begins by adding missing atoms (e.g., hydrogens) to the antigen as necessary and performing an energy relaxation in CHARMM [35]. The user may configure this relaxation when starting the experiment by indicating the number of CHARMM relaxation iterations. Following the relaxation, the antigen is rotated to minimize the *z*-coordinate of the epitope’s centroid (i.e., the mean of the coordinates of the epitope’s Cα atoms, neglecting atomic masses). This step orients the epitope towards the ensemble of MAPs parts that will be assembled into the variable domain, thus ensuring that the antibody will bind to the intended epitope. The implementation of a similar antigen rotation step in OptMAVEn has two significant limitations, which are corrected in OptMAVEn-2.0. First, OptMAVEn uses an exhaustive search of rotations around the *x* and *y* axes in discrete increments of 3° (i.e., 120 angles per axis yielding 120^2^ = 14,400 rotations) to minimize the *z*-coordinate of the epitope’s centroid. This search requires extensive sampling and typically lasts several minutes. Second, the search has a finite resolution (3° in each axis): the desired rotation may lie between two search points and thus may not be sampled. To illustrate, let the desired rotation *θ*_opt_ = (*θ_x_*_,opt_, *θ_y_*_,opt_) consist of a rotation around the *x* axis by *θ_x_*_,opt_ followed by a rotation around the *y* axis by *θ_y_*_,opt_. The discrete search will identify a point *θ*_opt_’ = (*θ_x_*_,opt_’, *θ_y_*_,opt_’) such that *θ_x_*_,opt_’, *θ_y_*_,opt_’ ∈ {0°, 3°, 6°, … , 357°}. The maximum difference between *θ*_opt_ and *θ*_opt_’ (for instance, if *θ*_opt_ = (1.5°, 1.5°)) is thus $\|\theta_{\mathrm{opt}}{}^{\prime }-\theta_{\mathrm{opt}}\|=\sqrt{1.5{{}^{\circ}}^{2}+1.5{{}^{\circ}}^{2}}=1.5{}^{\circ}\sqrt{2}\approx 2.1{}^{\circ}$. Thus, the final rotated antigen conformation in OptMAVEn may be up to 2.1° off with respect to the desired rotation.

OptMAVEn-2.0 corrects both problems by using a single matrix to perform the rotation. First, the centroids of the antigen ($\bar{c_{A}}$) and epitope ($\bar{c_{E}}$), and the vector between them $d=\bar{c_{E}}-\bar{c_{A}}$ are computed. Because the rotation does not change interatomic distances, ${\left\|d\right\|}^{2}=d_{x}{}^{2}+d_{y}{}^{2}+d_{z}{}^{2}$ remains unchanged during the rotation. Likewise, because $\bar{c_{A}}$ is the center of rotation, $\bar{c_{A}}$ must also remain unchanged. Thus, the rotation minimizes the *z* coordinate of the epitope’s centroid (${\bar{c_{E}}}_{z}$) subject to holding ${\left\|d\right\|}^{2}$ and $\bar{c_{A}}$ constant. Because $d_{x}{}^{2}+d_{y}{}^{2}\geq 0$, it must be true that $0\leq d_{z}{}^{2}\leq {\left\|d\right\|}^{2}$. Because $d_{z}{}^{2}={\left({\bar{c_{E}}}_{z}-{\bar{c_{A}}}_{z}\right)}^{2}$ and ${\bar{c_{A}}}_{z}$ is a constant, ${\bar{c_{E}}}_{z}$ may be decreased until the point at which ${\left({\bar{c_{E}}}_{z}-{\bar{c_{A}}}_{z}\right)}^{2}={\left\|d\right\|}^{2},d_{x}{}^{2}=d_{y}{}^{2}=0$. Thus, the solution that minimizes ${\bar{c_{E}}}_{z}$ is ${\bar{c_{E}}}_{x}={\bar{c_{A}}}_{x},{\bar{c_{E}}}_{y}={\bar{c_{A}}}_{y},{\bar{c_{E}}}_{z}={\bar{c_{A}}}_{z}-\left\|d\right\|$. This rotation is implemented using the *trans* procedure within Visual Molecular Dynamics (VMD) [36] software. If ‖d‖=0 (e.g., if all antigen residues are part of the epitope), no rotation is performed. This procedure outperforms OptMAVEn in that it requires no exhaustive search and yields an error of less than 0.01° in rotating the antigen such that the sum of the *z*-coordinates of its epitope is minimized.

**Figure 2:**
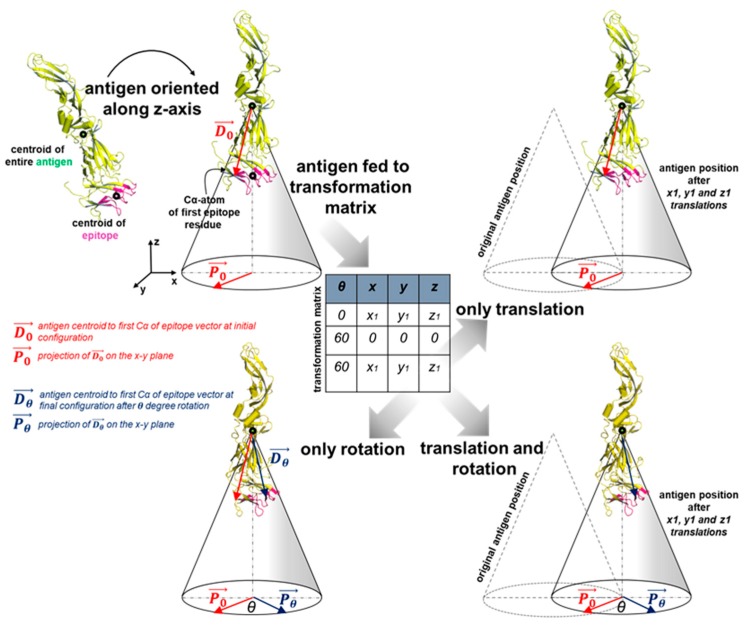
The grid search procedure. The antigen is first positioned such that (1) the centroid of its epitope is at the origin with the centroid of the antigen directly above it and (2) the *z*-rotation angle of the antigen (the angle between *P*_0_ and the positive *x* axis) is zero. An ensemble of positions of the antigen is generated by translating the centroid of the epitope or rotating the antigen around the *z* axis or both.

Following the rotation, OptMAVEn-2.0 generates an ensemble of antigen positions using a grid search (Figure 2). This step has been made significantly more efficient relative to OptMAVEn. An antigen-binding site is defined as the virtual box (obtained by inspecting 750 antigen-antibody binding regions) in which the *x*, *y*, and *z* coordinates of the epitope’s centroid are within the ranges [−10 Å, 5 Å], [−5 Å, 10 Å], and [3.75 Å, 16.25 Å], respectively. This box is partitioned into a grid (default *x*, *y*, and *z* intervals are 2.5, 2.5, and 1.25 Å, respectively). Furthermore, the antigen is rotated around the *z* axis to increase conformational sampling (the default is 6 rotations in increments of 60°). Hence, each antigen position can be represented as a so-called position vector consisting of the epitope centroid (*x*, *y*, and *z* coordinates) and the rotation angle around the *z* axis ($\theta_{z}$). The default settings lead to 6 × 7 × 7 × 11 = 3234 positions. OptMAVEn-2.0 introduces a precise definition of $\theta_{z}$ for peptide antigens, which was missing in OptMAVEn. Let $d_{1}=c_{1}-\bar{c_{A}}$ be the vector extending from the centroid of the antigen to the coordinate $c_{1}$ of the Cα atom of the first residue in the antigen. Then $\theta_{z}$ is defined as the angle between the positive *x*-unit vector ($\vec{ı}$) and the projection of $d_{1}$ onto the *x*-*y* plane. Using the relationship between angle and dot product, $\|\vec{ı}\|\|{\mathrm{proj}}_{x,y}\left(d_{1}\right)\|\cos\theta_{z}=\vec{ı}\cdot {\mathrm{proj}}_{x,y}\left(d_{1}\right)$, which leads to $\theta_{z}=\mathrm{sign}\left({\mathrm{proj}}_{x,y}{\left(d_{1}\right)}_{y}\right)\cdot \cos^{-1}\left(\frac{\vec{ı}\cdot {\mathrm{proj}}_{x,y}\left(d_{1}\right)}{\left\|\vec{ı}\|\|{\mathrm{proj}}_{x,y}\left(d_{1}\right)\right\|}\right)=\mathrm{sign}\left({\mathrm{proj}}_{x,y}{\left(d_{1}\right)}_{y}\right)\cdot \cos^{-1}\left(\frac{{\mathrm{proj}}_{x,y}{\left(d_{1}\right)}_{x}}{\left\|{\mathrm{proj}}_{x,y}\left(d_{1}\right)\right\|}\right)$.

As in OptMAVEn, OptMAVEn-2.0 screens out antigen positions that will inevitably lead to steric clashes with the representative structure of the antibody framework regions. Thus, antigen positions that clash with the framework will clash with any designed antibody and will yield energetically unfavorable designs. Herein, a position is defined as clashing if any atom of the antigen is within 1.25 Å of any atom in the framework. For each antigen position, the number of clashes is counted. While OptMAVEn tolerates up to two clashes, OptMAVEn-2.0 tolerates no clashes, as the former often resulted in interlocked aromatic side chains between residues of the epitope and the designed antibody structure.

OptMAVEn-2.0 significantly reduces disk storage requirements for antigen positioning by saving all non-clashing positions in a single text file (of a few kilobytes) and representing each as its position vector. Meanwhile, OptMAVEn saves each antigen conformation as its own PDB file. Since PDB files of large antigens can be of the order of several megabytes, alleviating the requirement to save thousands of PDB files could save gigabytes of storage. This choice contributes in large part to reducing the average maximum disk usage by 84%.

### 2.5. MAPs Interaction Energy Calculations

At each non-clashing antigen position, the interaction energy between the antigen and each MAPs part is calculated. OptMAVEn uses C++ modules that require a separate PDB file for each antigen position. However, OptMAVEn-2.0 implements the energy calculations by calling the NAMDEnergy [37] module of VMD, which is able to translate and rotate the antigen after loading its initial structure. Thus, we are able to generate all antigen positions using only a reference (starting) structure of the antigen and a second file of position vectors (prepared during the ‘Antigen positioning’ step), which together typically require only a few hundred kilobytes of disk space.

Both OptMAVEn and OptMAVEn-2.0 use electrostatic and van der Waals energy terms for choosing the optimal antibody parts during the MILP step. Full antibody variable domain designs emerging from the optimal MAPs parts selection step are re-optimized using an energy function that accounts for solvation effects. The binding scores thus calculated are now used to rank all the designs.

### 2.6. Optimal Selection of MAPs Parts

For each antigen position, OptMAVEn-2.0 selects one set of V, D, and J parts from the H locus and one set from either the K or L locus. It thereafter minimizes the sum of the interaction energies of the six parts using a mixed-integer linear program (MILP). In this program we define, set *I* = {*i* | HV, HCDR3, HJ, LV, LCDR3, LJ, KV, KCDR3, and K} that contains the nine categories of MAPs parts. Each category *i* has a set of part indexes *P_i_* = {*p* | 1, 2, …, N*_i_*}, where *N_i_* is the number of parts listed in category *i*. Each MAPs part is represented as a tuple (*i*, *p*) of a category and a serial index of that category. Further, the set *IP^clash^* = {((*i*_1_, *p*_1_), (*i*_2_, *p*_2_)), … ((*i*_m_, *p*_m_), (*i*_n_, *p*_n_))} is the set of all pairs of parts that sterically clash. The parameter *E_i_*_,*p*_ is the interaction energy between the antigen and MAPs part (*i*, *p*). The parameters *H_d_* and *L_d_* are set to 1 if the heavy and light variable domains, respectively, are being designed, and 0 otherwise. This allows the option of designing both domains (a full antibody) or a single domain (a nanobody). Finally, the binary variable *X_i_*_,*p*_ is equal to 1 if part (*i*, *p*) is chosen by the MILP to be a part of the final antibody design and is 0 otherwise. The optimization protocol uses an objective function subject to a set of five constraints as described below. The formulation is the same as that of OptMAVEn [16].

$$Minimize\overset{9}{\underset{i=1}{\sum }}\overset{N_{i}}{\underset{p=1}{\sum }}X_{i,p}E_{i,p}$$

subject to

(1) $$X_{i_{1},p_{1}}+X_{i_{2},p_{2}}\leq 1\;\forall \left\{\left(i_{1},p_{1}\right),\left(i_{2},p_{2}\right)\right\}\in IP^{clash}$$

(2) $$\overset{N_{i}}{\underset{p=1}{\sum }}X_{i,p}=H_{d},\;\forall i\in \left\{HV,HCDR3,HJ\right\}$$

(3) $$\overset{N_{KV}}{\underset{p=1}{\sum }}X_{KV,p}+\overset{N_{LV}}{\underset{p=1}{\sum }}X_{LV,p}=L_{d}$$

(4) $$\overset{N_{KV}}{\underset{p=1}{\sum }}X_{KV,p}=\overset{N_{KCDR3}}{\underset{p=1}{\sum }}X_{KCDR3,p}=\overset{N_{KJ}}{\underset{p=1}{\sum }}X_{KJ,p}$$

(5) $$\overset{N_{LV}}{\underset{p=1}{\sum }}X_{LV,p}=\overset{N_{LCDR3}}{\underset{p=1}{\sum }}X_{LCDR3,p}=\overset{N_{LJ}}{\underset{p=1}{\sum }}X_{LJ,p}$$

The objective function minimizes the interaction energy between the antigen and the set of MAPs parts that are selected. Constraint 1 prevents sterically clashing MAPs parts being chosen. Constraint 2 ensures that while a heavy chain is being designed, exactly one HV, HCDR3, and HJ part is selected, and that no heavy chain parts are selected if the heavy chain is not being designed (*H_d_* = 0). Constraint 3 is analogous to constraint 2 and ensures that if a KV part is selected, no LV parts are selected and vice versa. Constraint 4 ensures that if a KV part is chosen by constraint 3, one each of KCDR3 and KJ parts are also chosen, else no K chain parts should be chosen. Constraint 5 enforces the same for the L chain MAPs parts during the design. Together, constraints 3, 4, and 5 ensure that if a light chain is being designed, exactly one V, CDR3, and J part is selected for the light chain and prevent choosing a mix of kappa and lambda parts.

OptMAVEn-2.0 improves upon the design step of OptMAVEn in two ways. First, the *IP^clash^* set of OptMAVEn (48,800 pairs) was found to be incomplete, sometimes leading to designs with steric clashes between residues within the antibodies. Thus, despite having favorable interaction energies, these antibodies were structurally unstable. The current *IP^clash^* set has been updated to contain 66,604 additional pairs of MAPs parts and now identifies all pairs of parts for which any atom in one part is within 1 Å of any atom in the other (excluding pairs that cannot be selected simultaneously, such as HJ-1 and HJ-2 or LV-1 and KJ-1). A second improvement is that OptMAVEn-2.0 designs only one antibody for each antigen position, while OptMAVEn designed five. As the additional four antibodies designed by OptMAVEn were always sub-optimal to the first design, eliminating them would not eliminate the optimal design for each position. Moreover, the subsequent clustering step would likely cluster together designs at the same position but ultimately choose only or two designs from each cluster design, and so the last three or four designs at each position would very seldom, if at all, appear on the final list of the best designs. Thus, OptMAVEn-2.0 expends roughly one fifth of the effort during the design step without compromising the quality of the designs.

### 2.7. Antibody Assembly

OptMAVEn-2.0 creates a PDB file for each design by assembling the MAPs parts and positioning the antigen. These designs then undergo a structural relaxation (in CHARMM [35]) that first relieves any potential steric clashes and then uses van der Waals, electrostatics, and Generalized-Born solvation energy terms to calculate the antigen-antibody interaction energy. These interaction energies are used for the clustering step and subsequent ranking of all the designs.

### 2.8. Clustering the Antibody Designs

#### 2.8.1. Pre-Processing Step

OptMAVEn-2.0 clusters the antibody designs based on both their antigen positions (which are Euclidean coordinates) and the sets of MAPs parts they comprise (which are not Euclidean coordinates). To simultaneously cluster by position and MAPs parts, a Euclidean coordinate was generated for each MAPs part. Methods exist to compute distances between two biological sequences (e.g., the amino acid sequences of MAPs parts) [25] and to convert pairwise distance matrices into Euclidean coordinates only if (but not necessarily if) these distances satisfy the four criteria of a metric distance *d* [26]:

(6) d(x,y)≥0 ∀ x,y∈M

(7) d(x,y)=0⇔x=y ∀ x,y∈M

(8) d(x,y)=d(y,x) ∀ x,y∈M

(9)  d(x,y)+d(y,z)≥d(x,z) ∀ x,y,z∈M

where *x*, *y*, and *z* are sequences, *M* is a category of MAPs parts, and *d* is the function that computes a distance between two sequences. The first condition requires that all distances be positive, the second that two sequences have distance of zero if and only if they are identical, the third that the distance function is symmetric, and the fourth that the triangle inequality holds.

The method of Stojmirovic [26] is particularly well-suited to this task because it yields metric distances from biological sequences in the following manner. Let *s*(*x*, *y*) be a similarity score between sequences *x* and *y*, such that *s*(*x*, *y*) is greater if *x* and *y* are more similar. The associated quasi-metric distance *q* of the similarity score *s* is *q*(*x*, *y*) = *s*(*x*, *x*) − *s*(*x*, *y*). Finally, the distance *d*(*x*, *y*) = max{*q*(*x*, *y*), *q*(*y*, *x*)} is a metric, provided that s satisfies the following conditions [26]:

(10) s(x,x)≥s(x,y) ∀ x,y∈M

(11) s(x,x)=s(x,y)∧s(y,x)=s(y,y)⇒x=y ∀ x,y∈M

(12) s(x,y)+s(y,z)≤s(x,z)+s(y,y) ∀ x,y,z∈M

where *x*, *y*, and *z* are sequences and *M* is a category of MAPs parts. Most protein alignment scoring systems satisfy these conditions. Because the MAPs parts follow the international ImMunoGeneTics database (IMGT) numbering system [38], amino acids that have aligned with each other have the same residue number. Therefore, the similarity score between two sequences is the sum over all residue numbers of the alignment scores of the pair of aligned amino acids, or of a gap penalty if one sequence lacks a residue number.

$$s\left(x,y\right)=\underset{i\in A\cup^{\text{​}}B}{\sum }s^{\prime }\left(x_{i},y_{i}\right)$$

where *A* and *B* are the sets of residue numbers in sequences *x* and *y*, respectively; $x_{i}$ denotes the amino acid of number *i* in sequence *x* (or $x_{i}$ is a gap if i∉A); and $s^{\prime }\left(x_{i},y_{i}\right)$ is the similarity score between amino acids $x_{i}$ and $y_{i}$ in the BLOSUM62 matrix [39] if $i\in A\cap^{\text{​}}B$ or a gap penalty *g* otherwise. The optimal value of *g* was not known *a priori*, and so five levels (4, 6, 8, 10, and 12) were tested. For each level, we computed the similarity scores between all pairs of MAPs parts within every category and verified that they satisfied the conditions for *s*. Five violations of condition 2 revealed that there were five pairs of identical parts in the MAPs database: (HV-135, HV-136), (KV-2, KV-3), (KV-25, KV-26), (KV-41, KV-42), and (LV-5, LV-6). After removing the higher-numbered of the two parts from the database, all three conditions were satisfied.

**Figure 3:**
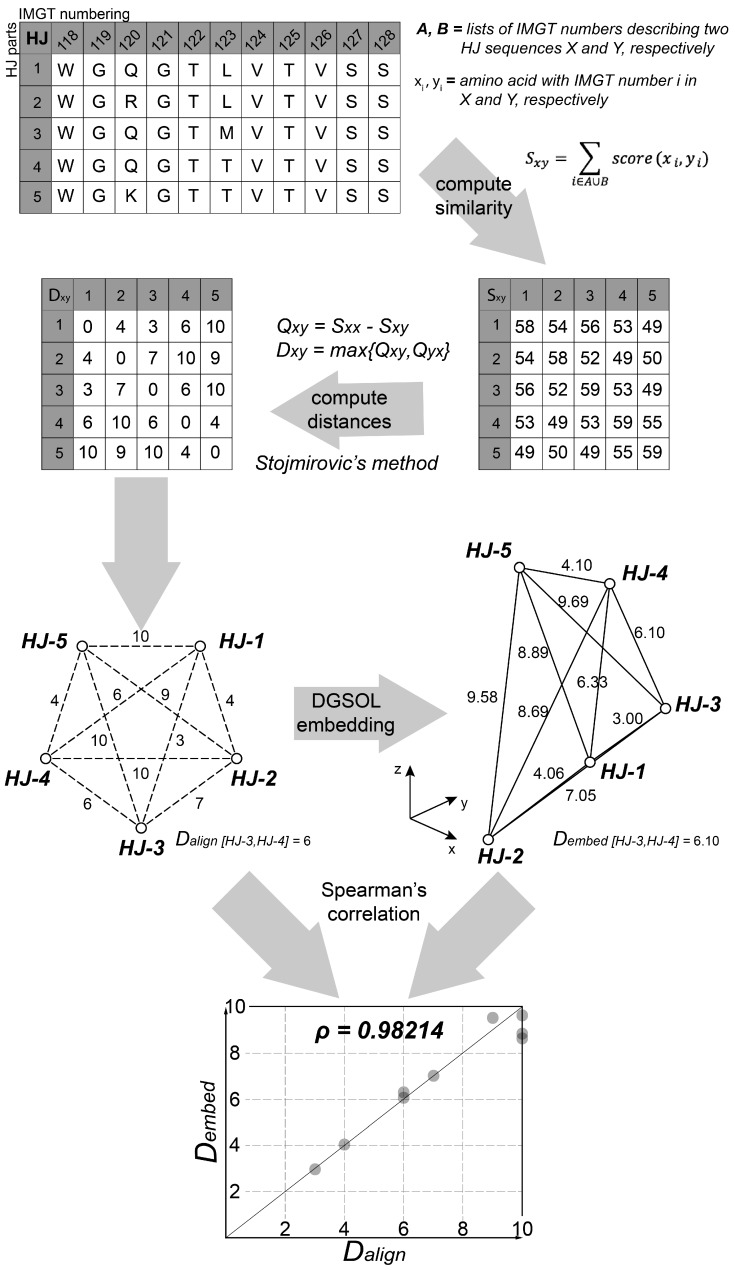
The steps involved in the embedder module. Actual values for HJ parts are provided as an example. First, the sequences are used to compute pairwise sequence similarity scores *S_xy_* using the BLOSUM62 matrix and a gap penalty *g*. From *S_xy_*, quasi-metric distances *Q_xy_* and their associated metric distances *D_xy_* are computed (e.g., *D_HJ-1_*_,*HJ-2*_ = 4). *D_xy_* can be visualized as a matrix or as a set of points and pairwise distances that cannot be embedded in Euclidean space. DGSOL generates Euclidean 3D coordinates for the points and computes the distances *D_embed_* between every pair of parts (e.g., *D_embed: HJ-1_*_,*HJ-2*_ = 4.06). It minimizes the sum of squared differences between corresponding aligned (*D_xy_*) and embedded (*D_embed_*) distances (e.g., [*D_HJ-1_*_,*HJ-2*_–*D_embed: HJ-1_*_,*HJ-2*_]^2^ = 0.0036). The Spearman rank correlation between *D_align_* and *D_embed_* is used to assess the quality of the embedding. If it is an abbreviation, please define.

Although the resulting pairwise distance matrix for each MAPs category satisfied the conditions for a metric, all such matrices possessed negative eigenvalues, indicating that they could not be embedded in Euclidean space [40]. Therefore, we devised a method to approximate a Euclidean embedding of these distances (Figure 3). Several programs—including MD-jeep [41], Xplor-NIH [42], TINKER [43], and DGSOL [27]—create approximate embeddings in 3D space. Although representing high-dimensional space in three dimensions causes the loss of some information, reducing the dimensionality helps to mitigate the so-called “curse of dimensionality” in the subsequent clustering step [44]. An attractive feature of DGSOL is that it accepts a lower and upper bound for each pairwise distance, enabling multiple sets of bounds to be tested. DGSOL computes a penalty function that depends on the extent to which the distances between embedded coordinates lie outside of the bounds; distances within the bounds are not penalized. The lower and upper bounds $LB_{ij}$ and $UB_{ij}$, respectively, were computed as $LB_{ij}=\left(1-w\right)\times d\left(x_{i},x_{j}\right)$ and $UB_{ij}=\left(1+w\right)\times d\left(x_{i},x_{j}\right)$ respectively, where $x_{i}$ and $x_{j}$ are two MAPs parts from the same category, *d* is the distance function, and *w* is a bound width parameter that was varied from 0.0 to 0.5 in increments of 0.05. For each level of *w* and of gap penalty *g*, DGSOL was used to generate an embedded coordinate $c_{i}$ for each MAPs part *i*. For each category of MAPs parts, the pairwise distances $c_{ij}=\|c_{i}-c_{j}\|$ between every pair of parts (i≥j) in the category were compared to the alignment distances from the $d_{ij}=d\left(x_{i},x_{j}\right)$ function. Specifically, the Spearman rank correlation ρ between $C=\{c_{ij}|i\geq j\}$ and $D=\{d_{ij}|i\geq j\}$ was calculated, as was the root mean square error $RMSE=\sqrt{\frac{\sum_{i\geq j}{\left(c_{ij}-d_{ij}\right)}^{2}}{N}}$, where *N* = card(*C*) = card(*D*) is the number of pairs of parts. The optimal *w* was chosen such that ρ was maximized. In the case of a tie, the *w* that minimized root mean squared error (RMSE) was chosen from among those *w* values that maximized ρ.

**Figure 4:**
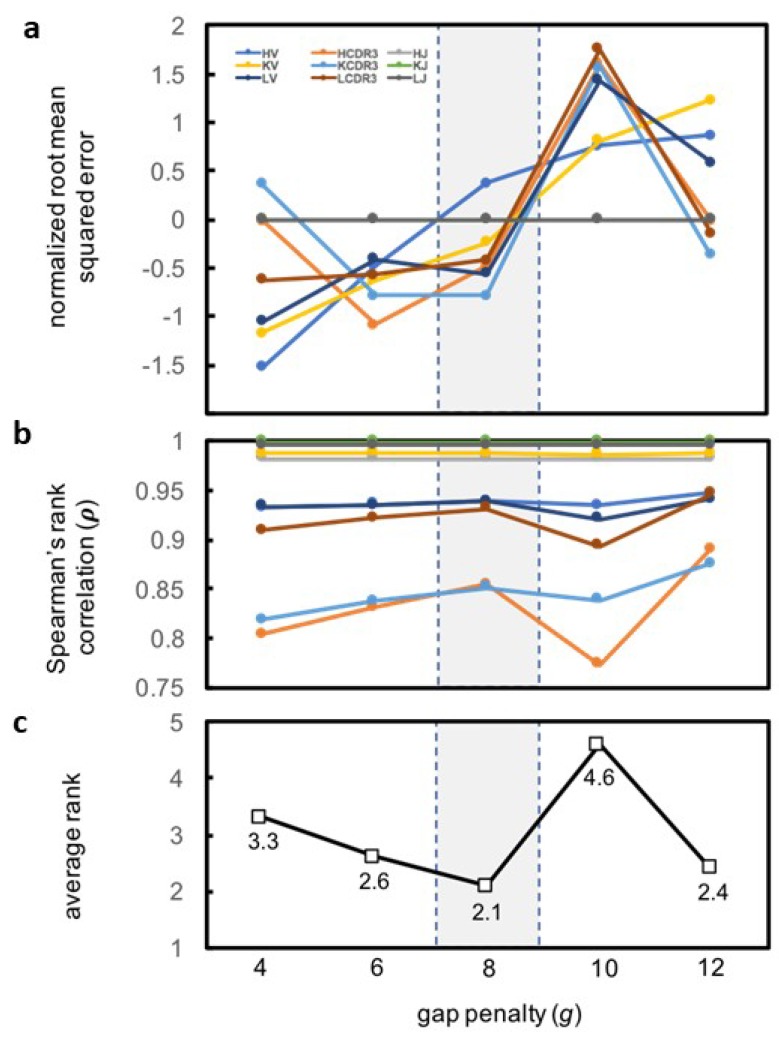
The optimal gap penalty (*g*) is 8. For each category of MAPs parts and each gap penalty *g* (4 to 12), pairwise aligned (*D_align_*) and embedded (*D_embed_*) distances were generated. (**a**,**b**) The z-score of *RMSE* between these distances the values of *ρ* were computed. Progressively increasing *g* led to a higher (desired) *ρ* and a higher (undesired) *RMSE z*-score with the exception of *g* = 10, which showed lower *ρ* and higher *RMSE z*-score than did *g* = 8; (**c**) The average rank of each *g* level for *ρ* and *RMSE* reveals *g* = 8 to be the best with an average rank 2.1.

The gap penalty *g* is used to compute sequence alignment distances $d_{ij}$, which are embedded and used to compute pairwise distances $c_{ij}$. Thus, ρ and *RMSE* (which depend on $d_{ij}$ and $c_{ij}$) depend on *g*. A ρ close to unity indicates that the relative order of distances was preserved during the embedding, and a *RMSE* close to zero indicates that the distances themselves were minimally perturbed. The optimal gap penalty (*g*) would maximize ρ and minimize *RMSE* for each MAPs category. To identify this optimal *g*, we tested five values of *g*: 4, 6, 8, 10, and 12. Each *g* was used to generate a similarity matrix *S* and an alignment matrix *D_align_* for each category of parts. The distances in *D*_align_ were embedded with DGSOL, and pairwise distances *D_embed_* between the embedded coordinates were computed. Then, ρ (Figure 4a and Supplementary Table S1) and *RMSE* (Figure 4b and Supplementary Table S2) were computed using *D_align_* and *D_embed_*. For each MAPs part category (HV, LV, and so on), we ranked the different *g* values in terms of the corresponding *ρ* (highest *ρ* yields rank 1 for the corresponding *g* and vice-versa) and of *RMSE* (lowest *RMSE* yields rank 1 for the corresponding *g* and vice-versa) (Table 1). Therefore, the rank of each *g* indicates how well the distances in *D_align_* could be embedded while preserving both relative and absolute distances. HJ, LJ, and KJ were excluded from this analysis because these parts contain no residue number gaps in the IMGT numbering; for these parts, *D_align_* and *D_embed_* do not depend on *g*. We found that *g* = 8 had the best average rank (2.1) (Figure 4c) and thus used *g* = 8 hereafter. However, the user has the option of selecting a different *g* from among the levels tested.

**Figure 5:**
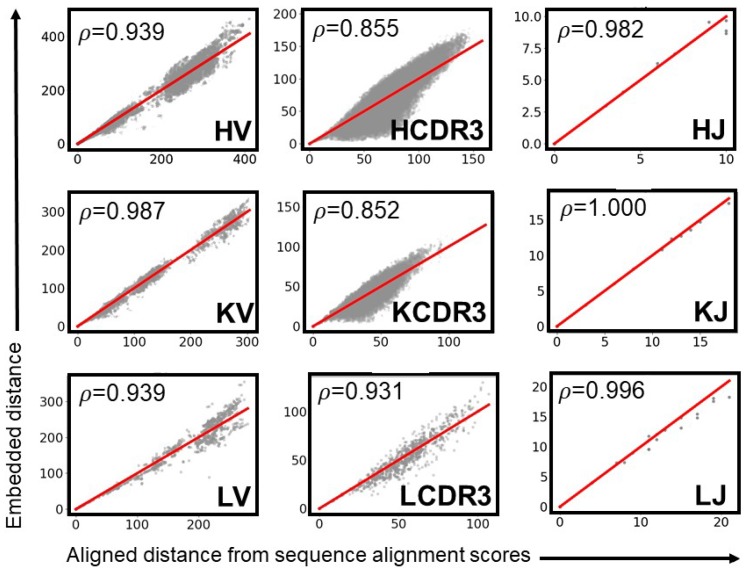
A 3D coordinate was computed for each MAPs part. For each pair of MAPs parts within each category, the two parts’ embedded distance in Euclidean space was plotted against their sequence alignment distance.

For *g* = 8, *ρ* was highest (*ρ* > 0.982) for the HJ, LJ, KJ and KV categories, showing that Euclidean coordinates recapitulated the relative ranks of the distances in *D_align_*. The CDR3 regions had the lowest values (0.851 < *ρ* < 0.932), indicating that the optimal Euclidean approximations swapped the ranks of a greater number of distances. Lower *ρ* values can presumably be attributed to the greater number of structures *N* in each CDR3 set (39 ≤ *N* ≤ 428) than in each J set (5 ≤ *N* ≤ 7). In the distance geometry problem, a set of pairwise distances between *N* points can be embedded into a Euclidean space of at most *N*–1 dimensions. Thus, the maximum potential dimensions of the spaces in which the CDR3 parts could be embedded are greater those of the spaces in which the J parts could be embedded. Projecting higher-dimensional coordinates onto 3 dimensions crushes more dimensions and thus causes more pairs of points that are far apart in high-dimensional space to become close together in three-dimensional space. Dimension crushing would create parts with large aligned distances but small embedded distances. Such parts appear most in the sets with the largest number of members (i.e., CDR3), less often in the medium-sized sets (i.e., V), and never in the smallest sets (i.e., J) (Figure 5).

#### 2.8.2. *k*-means Clustering

Each antigen position and its associated optimal set of MAPs parts is converted into a 23-dimensional vector by concatenating the *x*, *y*, and *z* coordinates of the epitope’s centroid; the sine and cosine of $\theta_{z}$; and the 3D coordinates representing the six MAPs parts. Clustering algorithms often fail to cluster high-dimensional data well due to the so-called “curse of dimensionality” [44]. Thus, the 23-dimensional vectors are normalized such that each dimension has unit variance (if the original variance is not zero), and PCA is performed to reduce the dimensionality of each vector to 3. Because the optimal number of clusters *k* is unknown prior to clustering, the clustering procedure initializes *k* to 1 and increments *k* after each round of clustering. During each round, the *k* clusters are initialized by randomly selecting (without replacement) one vector as the centroid for each cluster. Each vector is assigned to the cluster with the nearest centroid (measured by Euclidean distance). If any cluster is empty, a vector selected randomly from another cluster is moved to the empty cluster. Each cluster centroid is then moved to the geometric mean of the vectors in the cluster; the root mean square (RMS) movement is computed. The assignment and movement steps are repeated until the RMS movement falls below a threshold (default 0.01) or an iteration limit is reached (default 1000). For each cluster, the mean squared distance (MSD) between the centroid and the cluster members in computed; the maximum of these MSD values is assigned to the *k* value. For each *k*, the ratio of the MSD to the MSD for *k* = 1 is computed. The *k* value is incremented until this ratio falls below a threshold (default 0.2).

### 2.9. Ranking the Antibody Designs

OptMAVEn-2.0 ranks the designs using their clusters and their antigen-antibody interaction energies, ensuring that the highest-ranked designs are both structurally diverse and predicted to have high affinities.

Progressing from the cluster with the lowest to the cluster with the highest minimum energy, it collects the design with the minimum solvated interaction energy from each cluster and cycles back until all designs have been chosen. In this way, the most optimal design from every cluster is selected first, followed by the second-, third- and so forth most optimal designs.

The relaxed structure of each design is output as a PDB and a FASTA file in the directory OptMAVEn-2.0/experiments/name/antigen-antibody-complexes/Result_#. OptMAVEn-2.0 generates two additional files in the experiment’s directory. Summary.txt gives information about the experiment (e.g., antigen file, epitope). Results.csv lists all designs in descending order by rank and gives, for each, the antigen position, MAPs parts, antibody sequences, cluster number, and MILP, unrelaxed, and relaxed interaction energies.

## 3. Results

We first benchmarked OptMAVEn-2.0 against OptMAVEn with a set of 10 antigens and subsequently used 54 additional antigens to assess the performance of the current algorithm. We then used OptMAVEn-2.0 to design antibody variable fragments against two sets of Zika envelope proteins reported by Wang et al. [45] (PDB: 5GZN) and Zhao et al. [46] (5KVD, 5KVE, 5KVF, and 5KVG). We ranked our de novo designs along with the native antibody reported for 5GZN; 12 of 77 designs showed enhanced binding relative to the native. MD simulations performed on two out of these 12 designs showed that one design is stably bound to the antigen. Finally, we identified the key stabilizing antigen-antibody interactions in these two designs and the native antibody. Results from the second set of runs led to good native sequence recovery, with 55% of the top five de novo designed chains showing at least 50% identity and 40% of them showing 75% similarity.

Thereafter, we used OptMAVEn-2.0 to design antibodies against three lysozyme structures (1BVK [47], 4TSB [48], and 4PGJ [49]) for each of which there exists an experimentally reported humanized antibody that binds to it. We analyze the native sequence recovery from the top five best binding designs and also investigate the number of native epitope binding contacts that were also seen in the top five designs.

### 3.1. Computational Benchmarking of OptMAVEn and OptMAVEn-2.0 on 10 Antigens

OptMAVEn and OptMAVEn-2.0 were each used to design antibodies for a benchmarking set of 10 antigens (PDB codes: 1NSN, 2IGF, 2R0W, 2VXQ, 2ZUQ, 3BKY, 3FFD, 3G5V, 3L5W, and 3MLS). These antigens were selected randomly from the 120 antigens used to benchmark OptMAVEn [16]. The antigen chains and epitopes are given in Supplementary Table S3. Benchmarking was performed on a Linux InfiniBand cluster. We measured the amount of time taken for the steps of *Antigen Positioning* (*T*_pos_), *MAPs Interaction Energy Calculations* (*T*_ener_), and *Optimal Selection of MAPs Parts* (*T*_MILP_); as well as the maximum disk usage of the experiment directory (*D*_max_) for OptMAVEn (Table 2) and OptMAVEn-2.0 (Table 3). Time taken for the *k*-means clustering step could not be compared because this step is unique to OptMAVEn-2.0. Thus, total CPU time (*T*_CPU_) for purposes of comparison was defined as *T*_CPU_ = *T*_pos_ + *T*_ener_ + *T*_MILP_. We also recorded the number of positions that did not clash with the framework antibody (*N*_pos_) and the interaction energy (including Generalized Born solvation) of the most optimal antigen-antibody complex after structural relaxation with CHARMM (*E*_min_).

One potential confounding factor was that we used a different antigen binding site for OptMAVEn and OptMAVEn-2.0 during the antigen positioning step. In previous work [16], we used 750 antigen-antibody complexes from the Protein Data Bank to identify an antigen binding site of *x*: [−10 Å, 5 Å], *y*: [−5 Å, 10 Å], and *z*: [3.75 Å, 16.25 Å]. This binding site was used for OptMAVEn. During benchmarking of OptMAVEn-2.0, we interchanged the *x* and *y* dimensions of the binding site, that is *x*: [−5 Å, 10 Å], *y*: [−10 Å, 5 Å]. This change is not likely to have significantly affected *T*_CPU_, *D*_max_, or *N*_pos_ because it did not change the total number of grid points sampled (3234). However, this change would have affected *E*_min_ if the best design from OptMAVEn-2.0 was not within the original binding site of OptMAVEn—that is, if OptMAVEn could not have created the design. This was the case for only one antigen (2R0W) among the 10 tested; thus, we excluded 2R0W from the analysis of *E*_min_. There is no evidence that the difference in antigen binding sites confounded the results of OptMAVEn and OptMAVEn-2.0.

#### OptMAVEn-2.0 Reduces Time and Disk Requirements by 74% and 84%, Respectively

OptMAVEn-2.0 ran significantly faster than OptMAVEn in terms of *T*_CPU_ (mean 74% faster, *p* < 0.001), *T*_pos_ (mean 99.8% faster, *p* < 0.001), *T*_ener_ (mean 64% faster, *p* = 0.006), and *T*_MILP_ (mean 84% faster, *p* < 0.001). Additionally, average *D*_max_ was 84% lower for OptMAVEn-2.0 than for OptMAVEn (*p* < 0.001). These substantial improvements in performance did not compromise design quality: there was no significant difference in *E*_min_ between the two programs (*p* = 0.62) (Table 4). Because all quantities but *E*_min_ were ratios between OptMAVEn and OptMAVEn-2.0, we computed their *p* values using two-tailed ratio *t*-tests of log_10_(*Q/O*), where *Q* and *O* are the values for OptMAVEn-2.0 and OptMAVEn, respectively. The *p*-value for *E*_min_ was computed using a standard paired *t*-test of *Q*–*O*. We verified our assumptions of normality using Shapiro-Wilk tests: all *p*-values were >0.05.

### 3.2. Test of OptMAVEn-2.0 on 54 Additional Antigens Reveals Sub-Linear Scaling

In order to more fully analyze the relations between the performance metrics, we used OptMAVEn-2.0 to design antibodies (see Supplementary Table S4) for an additional 54 antigens (Table 5) that we selected randomly from the 120 antigens used to benchmark OptMAVEn. We found that *N*_pos_ correlated with both *T*_CPU_ (*r* = 0.663) and *D*_max_ (*r* = 0.954) more strongly than any other feature of the antigen correlated with these performance metrics. The number of residues (*N*_res_) or atoms (*N*_atom_) correlated only weakly with *T*_CPU_ (*r* = 0.083, *r* = 0.075, respectively). *N*_res_ and *N*_atom_ correlated moderately well with *D*_max_ (*r* = −0.472, *r* = −0.482, respectively) but, as larger antigens should require larger files, the negative sign was unexpected. Given the strong negative correlation between *N*_pos_ and *N*_atom_ (*r* = −0.650), it seems that larger antigens (measured by *N*_atom_) unsurprisingly tend to clash with the framework antibody in a larger number of positions and thus have lower *N*_pos_ values. Because *N*_pos_ is also the number of antibodies designed, decreasing *N*_pos_ reduces the number of files associated with antibody designs, decreasing *D*_max_. These results show that the computational resource requirements of OptMAVEn-2.0 scale in a sub-linear manner with the size of the antigen, *ceteris paribus*. Due to this feature, OptMAVEn-2.0 (unlike OptMAVEn) is capable of designing antibodies for very large antigens, e.g., Zika E protein (*N*_atom_ = 6801).

### 3.3. Test Cases on Zika E Protein

We used OptMAVEn-2.0 to design antibodies targeting epitopes of Zika E protein that we identified in the PDB entries 5GZN [45], 5KVD [46], 5KVE [46], 5KVF [46], and 5KVG [46]. While the antibodies in 5GZN are from a human, those in 5KVD, 5KVE, 5KVF, and 5KVG were raised in mice. The reported native antibody in each PDB binds Zika E protein with an affinity in the low nanomolar to low micromolar range. Unfortunately, we could not rank our de novo designs with respect to the native antibodies in 5KVD, 5KVE, 5KVF, and 5KVG because the native complexes are of poor quality, such that large steric clashes could not be alleviated even after several rounds of structural relaxations.

#### 3.3.1. Setup for the Test Cases on Zika E Protein

We defined an epitope residue such that at least one heavy atom of the residue was within 4 Å of at least one heavy atom of the antibody. The epitope residues are given in Supplementary Table S3. Note that if no structures of Zika in complex with an antibody had been available, we could have predicted these epitopes using existing software such as those described in Soria-Gurerra et al. [50]. We used the default settings for OptMAVEn-2.0 and defined the antigen binding box with the following bounds *x*: [−5 Å, 10 Å], *y*: [−10 Å, 5 Å], and *z*: [3.75 Å, 16.25 Å].

#### 3.3.2. Recovery of Native Residues in the Test Cases on Zika E Protein

We assessed the recovery of native residues by aligning each of the top five designs with the native sequence and computing % identity (identical residues) and % similarity (residues with similar properties) using EMBL EMBOSS Needle [51]. Native sequence recovery was reasonable (see Supplementary Table S1). Out of the 40 chains (20 heavy and 20 light chains from the top five designs of four cases), 22 (55%) chains were at least 50% identical, and 16 (40%) were at least 75% similar. Recovery of native L sequences was higher on average than that of H sequences: of the 22 chains that were at least 50% identical, 15 (68%) were L chains; and of the 16 chains that were at least 75% similar, 14 (88%) were L chains. This result likely arises because CDR-H3 is more diverse than CDR-L3.

#### 3.3.3. Humanization Scores in the Test Cases on Zika E Protein

We assessed the HScores of the top five designs and compared them to those of the native structure (see Table 6). The HScores of the de novo designs were consistently lower than those of the native antibody in all but two cases (5GZN light chain, 5KVF light chain, highlighted in bold). This result is unsurprising because all native antibodies but 5GZN are murine. Even relative to a human antibody (5GZN), the heavy chain HScores for the top five designs are consistently lower, which compensates for the relatively larger HScores of the light chains. The HScores suggest that OptMAVEn-2.0 can design antibodies with immunogenicities similar to those of human antibodies, although these predictions do not have experimental confirmation.

#### 3.3.4. Molecular Dynamics Simulations

We performed fast MD simulations using the QwikMD [29] protocol in VMD on three antibody-antigen complexes for 5GZN: the native complex, the top design (5gzn_R27, with the lowest interaction energy), and the design with the lowest MILP energy, which excludes solvation (5gzn_R0). The QwikMD trajectories were set up for 25 ns each of equilibration and production, with a time step of 2 fs; trajectory snapshots were kept every 1000 steps (2 ps). The simulations were run at 310 K with water as the implicit solvent.

**Figure 6:**
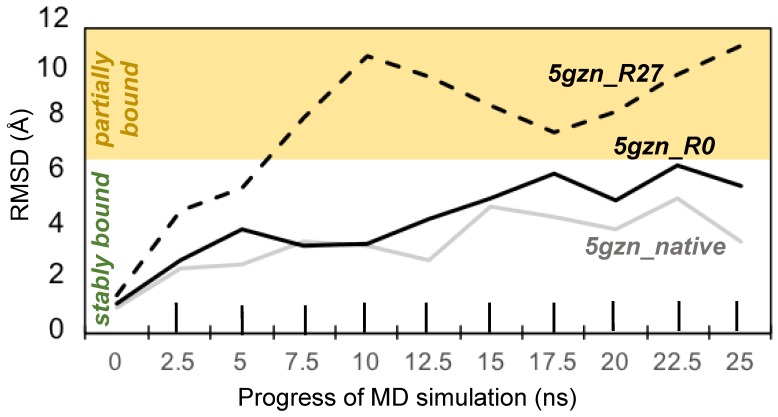
The native and 5gzn_R0 antigens remained stably bound (RMSD < 6 Å), while the 5gzn_R27 antigen remained partially bound (6 Å < RMSD < 12 Å) throughout the MD simulations. Heavy-atom RMSDs of antigen residues within a box at the antigen-antibody interface were computed after aligning the antibody residues within the box. The RMSDs for each complex are relative to the first frame (time 0 ns) of the production run.

**Figure 7:**
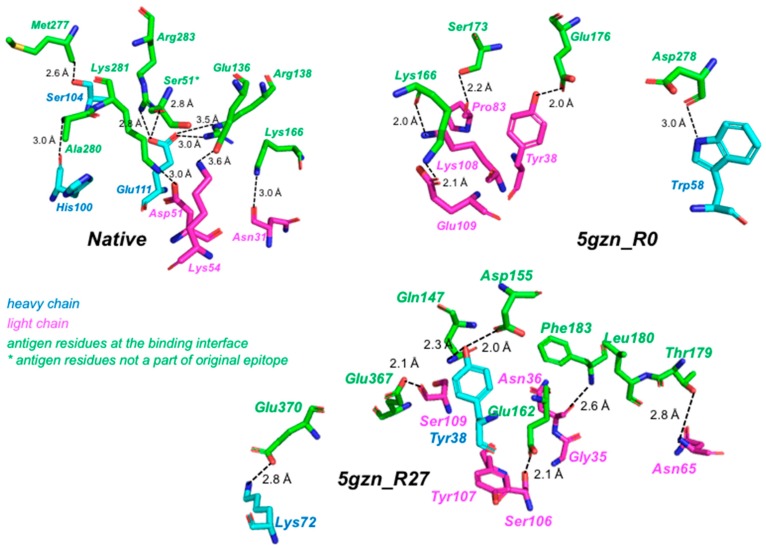
The key interactions at the antigen-antibody interface post-MD simulations for native, 5gzn_R0, and 5gzn_R27 have been depicted. The light and heavy chain residues are shown as magenta and cyan sticks, respectively, while antigen residues are depicted as green sticks.

We assessed the long-term stability of each of the three antigen-antibody complexes by calculating, once every 2.5 ns, the RMSD of the antigen with respect to the antigen at the beginning of the production run (i.e., time 0 ns). In order to analyze the stability of the antigen-antibody complex for the de novo designed antibody, we first identified the binding interface residues and tracked their fluctuations during the course of the 25 ns production run. Residues distal to the interface were neglected because unordered loop regions would contribute to larger root mean square deviations (RMSDs) even though the interface might be fairly stable. The antibody residues that are a part of the binding interface were aligned to their starting conformation (at 0 ns) at the end of every 2.5 ns of the 25 ns run. Then the heavy-atom RMSD of the antigen residues within the interface was computed (Figure 6). RMSDs of the native complex and 5 gzn_R0 were similar and remained below 6 Å in every frame examined, indicating that these complexes were stable throughout the entire simulations, according to a previous definition of stable binding by Poosarla et al. [52]. RMSD of 5gzn_R27 exceeded 6 Å but did not exceed 12 Å, indicating that the antigen remained partially bound [52]. Figure 7 shows the key electrostatic interactions (polar and salt bridge) seen in the 5gzn_native, 5gzn_R0, and 5gzn_R27 designs.

### 3.4. Test Cases on Hen Egg White Lysozyme

We identified three epitopes of hen egg white lysozyme from the PDB entries 1BVK [47], 4PGJ [49], and 4TSB [48]. The native antibodies in all three structures are human or humanized, though 4PGJ contains only the heavy chain in complex with lysozyme.

#### 3.4.1. Setup for the Test Cases on Lysozyme

We used the same definition of epitope residues as was used for Zika (see Table 2) and the default OptMAVEn-2.0 settings.

#### 3.4.2. Recovery of Native Residues and Contacts in the Test Cases on Lysozyme

For each test case, the sequences of the top five designs are given in Supplementary Figure S2. We assessed the recovery of native residues for these designs. Of the 20 chains designed for 1BVK and 4TSB, 18 (90%) are more than 65% similar and 9 (45%) are more than 75% similar. For 4PGJ, we found lower similarities in the range of 37–46%, likely because the native antibody was engineered using phage display with a library of humanized sequences, rather than isolated directly from a human. Excluding 4PGJ, 15 (75%) of the designed chains were at least 50% identical; the lowest identity observed was 40.7%, the highest 85.6%. These results show that OptMAVEn-2.0 can recover a high fraction of the residues in native human antibodies. We also report the percentage recovery of native antigen-antibody contacts in these top five designs (see Supplementary Figure S2) and their HScores in comparison to those of the native structures (see Table 7).

## 4. Summary and Discussion

OptMAVEn, an extension of the OptCDR framework, was the first software capable of designing entire variable domains de novo. However, OptMAVEn requires gigabytes of disk storage and weeks of CPU time, making it computationally intensive to target large antigens. We have developed OptMAVEn-2.0, which designs antibodies of equivalent affinities using significantly reduced disk storage (84% less) and CPU time (74% less). These improvements reduce the time needed to design germline antibodies from over a week to roughly one day and enable OptMAVEn-2.0 to handle large antigens, such as Zika E protein (407 residues) [45].

Due to its increased speed, OptMAVEn-2.0 could now be integrated into laboratory-based workflows for designing antibodies. The most common technologies for antibody development in the laboratory are animal immunization and phage display [13]. Immunization can yield low-affinity antibodies de novo in 1–2 weeks [53], while phage display can in some cases design high-affinity but non-specific antibodies in under one week and also requires an initial library of antigen binding fragments [54]. An integrated workflow would take advantage of the high affinities reached by phage display, as well as OptMAVEn-2.0′s speed (typically <24 h to design hundreds of variable domains) and abilities to minimize immunogenicity and target a specific epitope. Thus, we believe OptMAVEn-2.0 could enable the rapid design of candidate antibodies for experimental validation using only the antigen structure, unlike all other computational methods to our knowledge [16,17,18,19,55].

OptMAVEn-2.0 introduces a new clustering step that retains designs with high (unfavorable) interaction energies if they are the best designs among those with similar antigen poses and antibody sequences. Following the generation of germline designs, the designs can be validated with MD simulations (e.g., in QwikMD [29]). Designs that are likely to bind with high affinity according to the MD simulations can be further optimized using affinity maturation in IPRO [30], which increases affinity while lowering immunogenicity.

Despite these promising results, there are several limitations of OptMAVEn-2.0 on which we are currently working. As in OptMAVEn, the MILP step of OptMAVEn-2.0 still uses a simplified energy function that poorly estimates the chemical potential near the binding site; estimates worsen as the number of charged interactions increases. We have partially addressed this limitation by considering solvation when relaxing, clustering, and ranking the designs after the MILP based rotamer optimization step. Future work involves improving estimates of chemical potentials by incorporating solvation and entropy terms. Checa et al. [56] and Lazaridis and Karplus [57] have found that solvation energy contributions to protein-protein interactions are important. Solvation energy calculations could be further augmented by accounting for intramolecular self-solvation terms, as described by Choi et al. [58]. Additionally, incorporating the conformational entropy of the antigen would capture effects of unordered loops and binding site rotamers which are not held in place by a stable interaction with another residue, thereby providing meaningful insights about antigen-antibody binding biophysics [59].

Another limitation of OptMAVEn is that it does not explicitly consider the stability of the antibody itself. Antibodies are complex molecules and are prone to failure in multiple ways [60]. Aggregation of antibodies is a particular problem: when antibodies aggregate, they lose their ability to bind to the target ligand and increase the risk of becoming immunogenic [22], even for fully human antibodies [60]. Several methods have been developed to predict (e.g., Spatial Aggregation Propensity [22]) or remove (e.g., Rosetta Supercharge [23,24]) aggregation-prone regions of antibodies. Potentially, these tools or similar methods could be incorporated into the affinity maturation step of future versions of OptMAVEn. These methods would ensure that the aggregation risk did not increase during affinity maturation, just as the current implementation imposes a similar constraint on the HScore. Antibodies may also degrade chemically, such as through separation of the chains, oxidation, hydrolysis, or deamidation [60]. Future versions of OptMAVEn could include measures to reduce the risk of such degradation, thereby increasing shelf life or the tolerance of antibodies to a variety of conditions.

Currently, OptMAVEn-2.0 runs on the Institute of Cyber Science-Advanced CyberInfrastructure (ICS-ACI) cluster at Pennsylvania State University. In order to make it available to everyone without a CHARMM license, we plan to implement a web server on which users may submit jobs to be run. Like the command-line OptMAVEn-2.0 interface, this web server will prompt users for a structure file upload (or a PDB ID), the chain(s) in the antigen, and the epitope residues, as well as provide options to customize the settings of OptMAVEn-2.0. OptMAVEn-2.0 is freely available for download from both.

## 5. Conclusions

In this work, we have outlined an efficient protocol for rapid de novo design of antibody variable domains for specific antigen epitopes. OptMAVEn-2.0 is capable of designing and clustering antibodies under five hours for most antigen epitopes just using a single node and a single processor per node in ICS-ACI. A Humanizer module can be employed post OptMAVEn-2.0 to reduce immunogenicity with the objective of *in silico* affinity maturation. The Humanizer module can also be independently used for humanizing murine antibodies without having to go through the OptMAVEn-2.0 cascade. Both OptMAVEn-2.0 and Humanizer are freely available for download from both https://github.com/maranasgroup and http://www.maranasgroup.com/software.htm. We are currently working towards making both of these as web-based tools. However, currently a user can request for OptMAVEn-2.0 and Humanizer runs (http://www.maranasgroup.com/software.htm).

## Figures and Tables

**Table 1:** For each category of MAPs parts, the levels of the gap penalty *g* were ranked from 1 to 5 on the basis of *ρ* (highest *ρ* is rank 1) and RMSE (lowest *RMSE* is rank 1). J parts were excluded because for the J parts, *ρ* and *RMSE* are independent of *g*, as their sequences are devoid of residue gaps.

Category	Criterion	Gap Penalty (*g*)				
		Rank 1	Rank 2	Rank 3	Rank 4	Rank 5
HV	*ρ*	12	8	6	10	4
HCDR3	*ρ*	12	8	6	4	10
KV	*ρ*	12	8	6	4	10
KCDR3	*ρ*	12	8	10	6	4
LV	*ρ*	12	8	6	4	10
LCDR3	*ρ*	12	8	6	4	10
HV	RMSE	4	6	8	10	12
HCDR3	RMSE	6	8	12	4	10
KV	RMSE	4	8	6	12	10
KCDR3	RMSE	8	6	12	4	10
LV	RMSE	4	8	6	12	10
LCDR3	RMSE	4	6	8	12	10

**Table 2:** The performance of OptMAVEn on 10 antigens for benchmarking.

Antigen	*T*_pos_	*T*_ener_	*T*_MILP_	*T*_CPU_	*D*_max_	*E*_min_	*N*_pos_
1NSN	32.7	214.2	26.8	273.7	1004	−658.7	2428
2IGF	2.1	20.0	26.4	48.4	820	−76.4	3023
2R0W	2.0	17.8	20.2	40.0	779	−277.0 *	2955
2VXQ	26.1	174.4	19.6	220.1	970	−174.5	2711
2ZUQ	41.6	290.9	18.8	351.4	1094	−346.0	2645
3BKY	5.0	54.8	33.7	93.5	824	−216.1	3035
3FFD	5.3	35.0	19.5	59.8	657	+576.6	2347
3G5V	22.0	33.1	20.8	75.9	808	−309.9	2976
3L5W	29.6	173.9	24.4	227.9	1008	−281.4	2798
3MLS	5.8	53.0	21.9	80.7	809	−249.6	2903

*T*_pos_, *T*_ener_, *T*_MILP_, and *T*_CPU_ are in hours; *D*_max_ is in megabytes; *E*_min_ is the CHARMM binding energy score. * 2R0W was excluded from analysis of *E*_min_.

**Table 3:** The performance of OptMAVEn-2.0 on 10 antigens for benchmarking.

Antigen	*T*_pos_	*T*_ener_	*T*_MILP_	*T*_CPU_	*D*_max_	*E*_min_	*N*_pos_
1NSN	0.036	22.3	1.8	24.2	142.4	−438.1	442
2IGF	0.009	26.1	5.6	31.7	169.7	−118.5	1374
2R0W	0.010	22.4	4.9	27.4	152.9	−127.9 *	1204
2VXQ	0.033	33.7	3.6	37.4	135.4	−235.3	893
2ZUQ	0.046	40.4	3.2	43.6	167.3	−131.3	774
3BKY	0.011	33.9	6.7	40.6	197.4	−208.4	1647
3FFD	0.014	10.9	2.0	13.0	83.8	+92.6	492
3G5V	0.012	21.0	4.2	25.2	137.6	−458.5	1035
3L5W	0.033	36.4	3.8	40.2	144.7	−394.0	910
3MLS	0.009	18.0	3.3	21.3	114.7	−171.2	807

*Tpos, Tener, TMILP,* and *TCPU* are in hours; *Dmax* is in megabytes; *Emin* is the CHARMM binding energy score. * 2R0W was excluded from analysis of *Emin.*

**Table 4:** Comparison of the performances of OptMAVEn and OptMAVEn-2.0 on 10 antigens.

Antigen	*T*_pos_	*T*_ener_	*T*_MILP_	*T*_CPU_	*D*_max_	*E*_min_	*N*_pos_
1NSN	−2.96	−0.982	−1.162	−1.053	−0.848	+220.6	−0.740
2IGF	−2.35	+0.116	−0.674	−0.184	−0.684	−42.1	−0.342
2R0W	−2.29	+0.102	−0.613	−0.165	−0.707	+149.1 *	−0.390
2VXQ	−2.90	−0.714	−0.732	−0.770	−0.855	−60.8	−0.482
2ZUQ	−2.95	−0.857	−0.774	−0.906	−0.815	+214.6	−0.534
3BKY	−2.65	−0.208	−0.700	−0.362	−0.620	+7.7	−0.265
3FFD	−2.58	−0.505	−0.984	−0.663	−0.895	−484.0	−0.679
3G5V	−3.27	−0.198	−0.698	−0.479	−0.769	−148.6	−0.459
3L5W	−2.95	−0.680	−0.806	−0.753	−0.843	−112.6	−0.488
3MLS	−2.80	−0.469	−0.823	−0.578	−0.848	+78.4	−0.556
Shapiro *P*	6.0 × 10^−1^	5.8 × 10^−1^	1.0 × 10^−1^	8.2 × 10^−1^	1.8 × 10^−1^	3.6 × 10^−1^	9.4 × 10^−1^
mean	−2.77	−0.440	−0.797	−0.591	−0.788	−36.3	−0.494
s. d.	0.303	0.383	0.164	0.296	0.090	213.2	0.145
*p*-value	**3.5 × 10^−10^**	**5.5 × 10^−3^**	**9.2 × 10^−8^**	**1.4 × 10^−4^**	**5.0 × 10^−10^**	**6.2 × 10^−1^**	**1.9 × 10^−6^**
mean (ratio)	0.002	0.363	0.160	0.256	0.163	n/a	0.321
% reduction	99.8	63.7	84.0	74.4	83.7	n/a	67.9

*T*_pos_, *T*_ener_, *T*_MILP_, *T*_CPU_, D_max_, and *N*_pos_ report the log_10_ of the ratios of the corresponding OptMAVEn-2.0 and OptMAVEn values. *E*_min_ reports the difference of the corresponding OptMAVEn-2.0 and OptMAVEn values. The Shapiro-Wilk test shows that every set of values is close to normal (*p* > 0.05). OptMAVEn-2.0 performed significantly better (*p*-value < 0.05) in *T*_pos_, *T*_ener_, *T*_MILP_, *T*_CPU_, and *D*_max_ and yielded designs of equivalent *E*_min_ (*p*-value = 0.79). The mean (ratio) gives, for the quantities reported as log_10_ ratios, the value of the mean ratio (i.e., 10^mean^). The % reduction is 100%–mean (ratio). * 2R0W was excluded from analysis of *E*_min_.

**Table 5:** OptMAVEn-2.0 was tested on 54 antigens in addition to those used for benchmarking against OptMAVEn.

Antigen	*N*_res_	*N*_atom_	*N*_pos_	*T*_CPU_	*D*_max_	*E*_min_
1ACY	10	156	1558	40.9	188.3	−370.6
1CE1	8	93	1694	44.3	200.9	−513.3
1CFT	5	84	1554	38.9	187.9	−253.5
1DZB	129	1958	749	42.2	136.3	−775.8
1EGJ	101	1643	650	34.1	106.9	−618.6
1F90	9	156	1328	35.0	165.8	−377.5
1FPT	11	162	1478	38.4	180.0	−455.6
1HH6	11	159	718	20.8	104.6	−385.5
1I8I	9	142	1480	38.4	179.7	−350.8
1JHL	129	1962	985	53.7	132.3	−766.6
1JRH	95	1491	397	21.9	99.6	−541.4
1KC5	8	119	1299	36.8	162.1	−376.1
1KIQ	129	1968	730	41.4	119.1	−750.1
1MLC	129	1968	618	35.9	111.2	−752.0
1N64	16	241	990	28.1	132.9	−386.6
1NAK	10	166	1192	41.5	154.1	−393.3
1OBE	13	195	417	13.5	77.9	−397.0
1ORS	132	2146	1001	55.7	162.4	−625.5
1PZ5	8	124	1348	34.1	167.4	−419.5
1QNZ	18	301	575	18.5	91.4	−367.3
1SM3	9	126	1354	34.8	167.9	−454.2
1TQB	102	1659	489	26.8	104.1	−534.6
1V7M	145	2258	588	37.5	115.4	−561.0
1XGY	6	85	1811	45.4	212.8	−293.1
1ZA3	91	1346	71	7.5	91.8	−758.7
2A6I	9	136	1093	29.1	141.8	−365.2
2BDN	68	1106	810	35.2	115.1	−740.6
2DQJ	129	1968	590	34.0	111.6	−852.4
2FJH	98	1565	312	18.4	99.7	−528.8
2H1P	11	182	561	17.0	90.4	−355.0
2HH0	9	151	1062	28.6	140.0	−282.7
2HRP	10	177	1013	27.9	135.4	−366.5
2IFF	129	1966	595	33.9	126.7	−594.4
2JEL	85	1293	596	28.1	101.9	−539.5
2OR9	11	181	734	21.1	106.7	−387.8
2QHR	11	185	761	20.3	111.3	−340.2
2R29	97	1553	641	33.2	105.3	−698.4
3AB0	136	1955	380	23.9	107.8	−765.0
3BDY	95	1521	779	36.3	133.9	−439.7
3CVH	8	142	1168	30.7	149.6	−333.7
3D85	133	2074	441	27.9	109.8	−717.0
3E8U	11	136	1481	38.1	180.8	−431.4
3ETB	144	2332	296	21.8	111.3	−898.6
3F58	11	136	1317	34.6	168.5	−322.6
3G6D	106	1667	418	24.2	103.2	−876.8
3GHB	10	146	1341	33.5	166.7	−383.4
3GHE	15	255	773	26.9	112.2	−430.1
3HR5	9	142	1340	38.4	166.5	−478.7
3KS0	92	1443	1148	54.3	148.0	−578.5
3MLX	14	235	621	20.5	94.7	−367.7
3NFP	124	1909	292	19.7	104.5	−771.6
3P30	84	1437	32	4.7	65.2	−714.9
3QG6	6	105	1425	36.1	175.2	−362.4
3RKD	146	2185	776	46.1	124.5	−793.7

*T*_CPU_ is in hours, *D*_max_ is in megabytes, and *E*_min_ is the CHARMM binding energy score.

**Table 6:** Comparison of HScores of the top five de novo designs with the HScores of the native antibodies.

Accession	Antibody Name (from Paper)	Native Heavy Chain HScore	Designed Heavy Chain HScores	Native Light Chain HScore	Designed Light Chain HScores
5GZN	Z3L1	52	17–36	4	16–41
5KVD	ZV-2	152	6–59	56	0–31
5KVE	ZV-48	128	21–68	59	1–27
5KVF	ZV-64	107	21–44	22	22–30
5KVG	ZV-67	133	10–39	111	10–25

**Table 7:** Comparison of HScores of the top five de novo designs with the HScores of the native antibodies.

Accession	Native Heavy Chain HScore	Designed Heavy Chain HScores	Native Light Chain HScore	Designed Light Chain HScores
1BVK	85	10–37	57	7–27
4TSB	26	12–32	21	16–38
4PGJ	87	20–49	N/A	5–39

## Supplementary Materials

The following are available online at http://www.mdpi.com/2073-4468/7/3/23/s1, Figure S1: Sequence alignments of the native heavy and light chain antibody sequences with the top five de novo designed sequences for (a) 5KVD, (b) 5KVE, (c) 5KVF, and (d) 5KVG respectively, have been represented, Figure S2: Sequence alignments of the native heavy and light chain antibody sequences with the top five de novo designed sequences for (a) 1BVK, (b) 4TSB, and (c) 4PGJ have been represented, Table S1: The Spearman rank correlation coefficient (ρ) for each MAPs part category at each gap penalty g, Table S2: The root mean squared error (RMSE) for each MAPs part category at each gap penalty g, Table S3: The antigen chain, heavy chain, light chain, and epitope residues from each of the 64 antigens used for testing OptMAVEn-2.0, Table S4: The antigen chains and epitope residues of the designs used in the test cases.
