# Supplementary material for: OptMAVEn-2.0: De novo Design of Variable Antibody Regions against Targeted Antigen Epitopes
Source: Antibodies (Basel). 2018 Jun 30;7(3):23. doi: 10.3390/antib7030023 (PMC6640672; doi:10.3390/antib7030023)
Supplement: Supplementary file 1 [file antibodies-07-00023-s001.pdf]

<u>Supporting Information:</u> OptMAVEn-2.0: *De novo* design of variable antibody regions against targeted antigen epitopes

Ratul Chowdhury[1,§], Matthew F. Allan[2,§], & Costas D. Maranas[1,*]

[1]Department of Chemical Engineering, The Pennsylvania State University, University Park, PA 16802. USA.
[2]Department of Computational and Systems Biology, Massachusetts Institute of Technology, Cambridge, MA 02139. USA.

[§]These authors contributed equally to this work

* Corresponding author

Table S1. The Spearman rank correlation coefficient (ρ) for each MAPs part category at each gap penalty g. ρ is independent of g for the J parts because the J parts do not have gaps.

g	HV	HCDR3	HJ	LV	LCDR3	LJ	KV	KCDR3	KJ
4	0.932	0.804	0.982	0.987	0.818	1.000	0.934	0.910	0.996
6	0.935	0.831	0.982	0.988	0.838	1.000	0.935	0.922	0.996
8	0.939	0.855	0.982	0.987	0.852	1.000	0.939	0.931	0.996
10	0.935	0.774	0.982	0.986	0.839	1.000	0.921	0.894	0.996
12	0.948	0.891	0.982	0.987	0.875	1.000	0.941	0.946	0.996

Table S2. The root mean squared error (*RMSE*) for each MAPs part category at each gap penalty g. *RMSE* is independent of g for the J parts because the J parts do not have gaps.

g	HV	HCDR3	HJ	LV	LCDR3	LJ	KV	KCDR3	KJ
4	26.313	15.441	0.593	13.005	9.833	0.321	21.669	8.484	1.124
6	26.793	15.133	0.593	13.146	9.585	0.321	21.983	8.508	1.124
8	27.179	15.306	0.593	13.244	9.585	0.321	21.906	8.574	1.124
10	27.360	15.902	0.593	13.513	10.096	0.321	22.877	9.551	1.124
12	27.404	15.439	0.593	13.620	9.674	0.321	22.468	8.699	1.124

Table S3. The antigen chain, heavy chain, light chain, and epitope residues from each of the 64 antigens used for testing OptMAVEn-2.0. The ten antigens used for benchmarking are tabulated first.

PDB	Antigen Chain	Heavy Chain	Light Chain	Epitope
1NSN	S	H	L	57, 60, 61, 64, 68, 70, 93, 95, 96, 97, 98, 105, 106, 120, 121, 123, 124, 127
2IGF	P	H	L	70, 71, 72, 73, 74, 75
2R0W	Q	H	L	3, 4, 5, 6, 7, 8
2VXQ	A	H	L	32, 34, 39, 40, 41, 43, 67, 68, 69, 74, 75, 76, 77, 78, 79, 80
2ZUQ	A	C	B	95, 96, 97, 98, 99, 100, 101, 132, 133, 134, 141

3BKY	P	H	L	169, 170, 171, 172, 173, 174, 175
3FFD	P	A	B	16, 17, 19, 20, 21, 23, 24, 26, 27, 28, 30, 31
3G5V	C	B	A	288, 289, 293, 296, 297, 298, 299, 300, 301, 302
3L5W	I	H	L	14, 15, 101, 104, 105, 107, 108, 109
3MLS	P	H	L	3, 5, 6, 10, 11, 12, 13, 14, 15, 16, 17, 18
1ACY	P	H	L	316, 319, 320, 321, 322, 323, 324
1CE1	P	H	L	0, 1, 2, 3, 4, 5, 6, 7
1CFT	C	B	A	1, 2, 3, 4, 5
1DZB	X	A	A	20, 21, 23, 62, 63, 73, 75, 96, 97, 98, 100, 101, 102, 103, 104, 106, 112, 116
1EGJ	A	H	L	362, 363, 364, 365, 366, 367, 395, 416, 417, 418, 419, 421
1F90	E	H	L	1, 2, 3, 4, 5, 6, 7, 8
1FPT	P	H	L	96, 97, 98, 99, 100, 101, 102, 103
1HH6	C	B	A	2, 3, 4, 5, 6, 7, 8, 9, 10, 11
1I8I	C	B	A	502, 503, 505, 506, 507, 508, 509
1JHL	A	H	L	21, 22, 23, 102, 103, 104, 106, 111, 112, 113, 116, 117, 118, 119, 121
1JRH	I	H	L	47, 49, 50, 51, 52, 53, 54, 55, 56, 76, 78, 79, 80, 82, 84, 98, 99
1KC5	P	H	L	1, 2, 3, 4, 5, 6, 7
1KIQ	C	B	A	18, 19, 22, 23, 24, 25, 27, 102, 103, 116, 117, 118, 119, 120, 121, 124, 125
1MLC	E	B	A	41, 43, 45, 46, 47, 48, 49, 50, 51, 53, 66, 67, 68, 70, 79, 81, 84
1N64	P	H	L	26, 29, 30, 31, 32, 33, 34, 35, 36, 37, 38
1NAK	P	H	L	313, 314, 315, 316, 319, 320, 321, 322
1OBE	P	H	L	1, 2, 3, 4, 5, 6, 7, 8, 9, 10, 11, 12
1ORS	C	B	A	111, 112, 113, 114, 115, 116, 117, 119, 120, 123
1PZ5	C	B	A	1, 2, 3, 4, 5, 6, 7, 8
1QNZ	P	H	L	233, 234, 235, 236, 237, 238, 239, 240, 241, 242, 243, 244, 245, 248
1SM3	P	H	L	2, 3, 4, 5, 6, 7, 8, 9, 10
1TQB	A	B	C	127, 128, 158, 159, 185, 188, 189, 190, 191, 192, 193, 194, 195, 196, 197, 198, 199
1V7M	V	H	L	57, 58, 61, 68, 71, 75, 98, 101, 102, 105, 106, 109, 110, 111, 112, 113, 114
1XGY	P	H	L	1, 2, 3, 4, 5, 6
1ZA3	R	H	L	25, 26, 27, 34, 36, 37, 38, 53, 54, 56, 57, 58, 59, 61, 62
2A6I	P	B	A	3, 4, 5, 6, 7, 8, 9, 10
2BDN	A	H	L	28, 30, 31, 32, 34, 37, 38, 39, 40, 41, 55, 56, 61, 64, 65, 68, 69
2DQJ	Y	H	L	14, 15, 16, 19, 20, 21, 62, 63, 73, 74, 75, 77, 93, 96, 97, 98, 100, 101, 102, 103
2FJH	V	H	L	16, 17, 18, 19, 21, 22, 23, 25, 61, 66, 101, 104
2H1P	P	H	L	602, 603, 604, 605, 606, 608, 609, 610, 611, 612
2HH0	P	H	L	2, 3, 4, 5, 6, 7, 8, 9, 10
2HRP	P	H	L	36, 37, 38, 39, 40, 41, 42, 43, 44, 45

2IFF	Y	H	L	41, 43, 45, 46, 47, 48, 49, 51, 53, 67, 68, 69, 70, 81, 84
2JEL	P	H	L	1, 2, 3, 4, 34, 36, 41, 64, 66, 67, 68, 70, 71, 72, 75, 76
2OR9	P	H	L	1, 2, 3, 4, 5, 6, 7, 8, 9, 10
2QHR	P	H	L	405, 406, 407, 408, 409, 410, 411, 412, 413, 414
2R29	A	H	L	306, 307, 308, 309, 310, 311, 312, 325, 362, 363, 364, 387, 388, 389, 390, 391
3AB0	A	B	C	102, 104, 113, 119, 120, 121, 122, 123, 125, 154, 156, 157, 158, 186, 188, 190, 192
3BDY	V	H	L	48, 81, 83, 84, 85, 86, 87, 88, 89, 90, 91, 92, 93
3CVH	C	H	L	4, 5, 6, 7, 8
3D85	C	B	A	82, 86, 87, 88, 91, 92, 93, 94, 95, 106, 110, 133, 134, 135, 136, 137, 139, 140
3E8U	P	H	L	4, 5, 6, 7, 8, 9, 10, 11, 13
3ETB	J	H	L	649, 651, 652, 653, 654, 655, 657, 680, 681, 682, 683, 684, 685, 686, 687, 716, 718
3F58	P	H	L	315, 316, 319, 320, 321, 322, 323, 324, 10, 11
3G6D	A	H	L	5, 6, 14, 18, 19, 21, 22, 23, 24, 97, 100, 101, 104, 105, 107, 108, 109
3GHB	P	H	L	306, 307, 308, 309, 312, 313, 314, 315
3GHE	P	H	L	304, 305, 306, 307, 308, 309, 312, 313, 314, 315, 316
3HR5	R	H	L	7, 8, 9, 10, 11, 12, 13, 14, 15
3KS0	B	H	L	30, 63, 64, 65, 66, 67, 68, 69, 70, 72, 74
3MLX	P	H	L	305, 306, 307, 308, 309, 312, 313, 314, 315, 316, 317, 318, 319
3NFP	I	H	L	1, 2, 3, 4, 5, 6, 25, 27, 43, 45, 116, 118, 120, 149, 152, 153, 154, 155
3P30	A	H	L	640, 643, 647, 650, 651, 653, 654, 657, 658, 661
3QG6	C	H	L	3, 4, 5, 6, 7, 8
3RKD	A	H	L	476, 477, 479, 484, 485, 496, 497, 498, 499, 508, 510, 512, 513, 514, 515, 534, 572, 573, 574, 575, 576, 577, 578, 592

Table S4. The antigen chains and epitope residues of the designs used in the test cases.

PDB	Antibody	Chain	Epitope residues
5GZN	Z3L1	A	46, 47, 52, 136, 138, 140, 156, 158, 159, 166, 168, 276, 277, 278, 279, 280, 281, 283
5KVD	ZV-2	E	301, 315, 316, 317, 318, 319, 320, 321, 322, 327, 329, 362, 364, 365, 366, 367, 372, 373, 374, 375, 377
5KVE	ZV-48	E	307, 340, 342, 343, 344, 347, 348, 350, 351, 352, 353, 354, 355, 358, 384, 386
5KVF	ZV-64	E	307, 340, 342, 343, 344, 347, 348, 350, 351, 352, 353, 354, 355, 358, 391
5KVG	ZV-67	E	309, 310, 311, 312, 313, 314, 331, 332, 333, 334, 335, 336, 337, 368, 370, 371, 393, 394, 395, 396, 397
1BVK	N/A	C	18, 19, 22, 23, 24, 27, 102, 103, 116, 117, 118, 119, 120, 121, 124, 125, 129
4PGJ	N/A	B	35, 46, 47, 48, 49, 52, 57, 59, 61, 62, 63, 70, 107, 108, 109, 110, 112
4TSB	N/A	A	21, 22, 23, 102, 103, 104, 106, 109, 111, 112, 113, 114, 116, 117, 118

Supplementary Figure 1:.

(a)

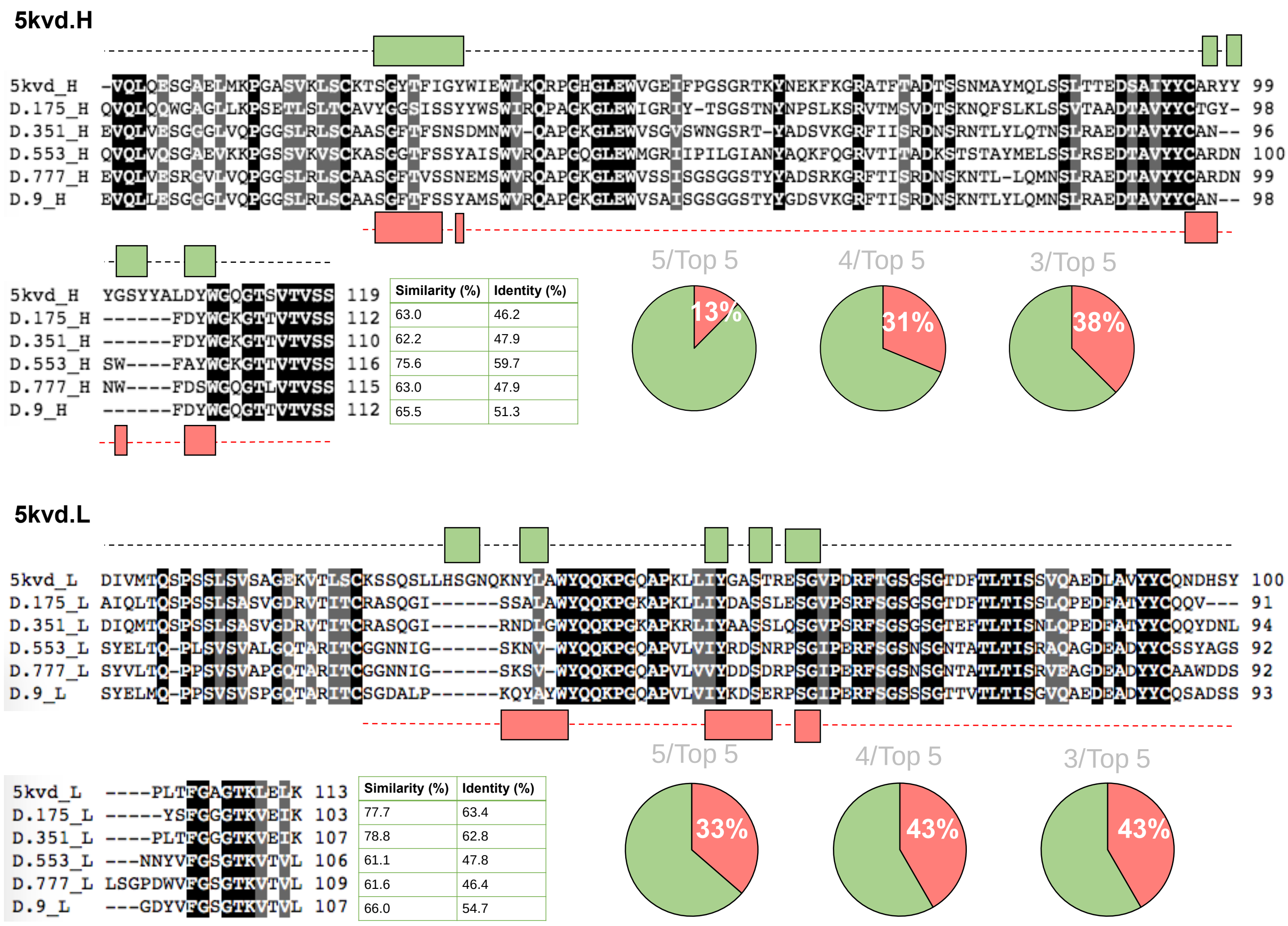

(b)

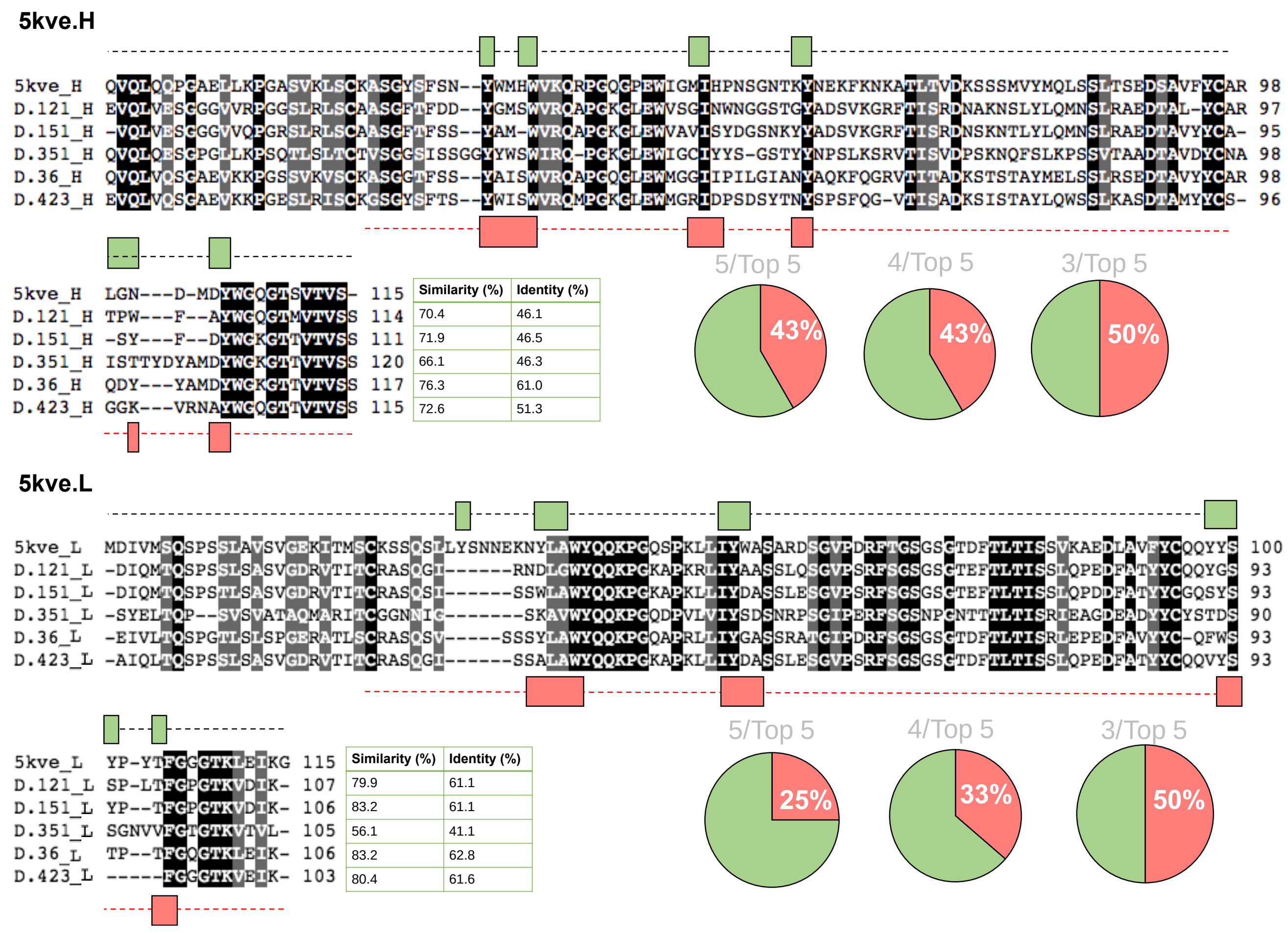

Similarity (%)	Identity (%)
70.4	46.1
71.9	46.5
66.1	46.3
76.3	61.0
72.6	51.3

Similarity (%)	Identity (%)
79.9	61.1
83.2	61.1
56.1	41.1
83.2	62.8
80.4	61.6

(c)

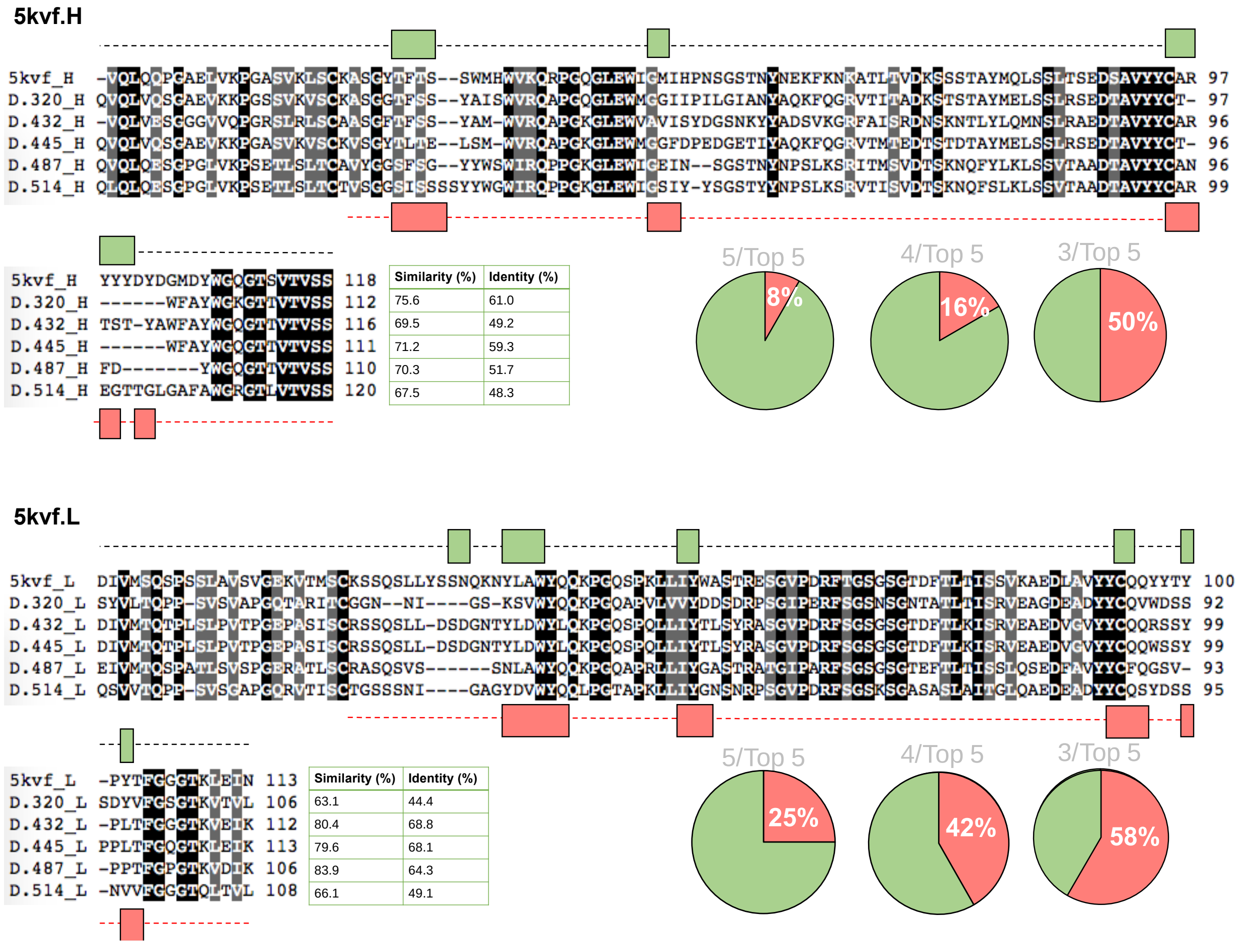

Similarity (%)	Identity (%)
75.6	61.0
69.5	49.2
71.2	59.3
70.3	51.7
67.5	48.3

Similarity (%)	Identity (%)
63.1	44.4
80.4	68.8
79.6	68.1
83.9	64.3
66.1	49.1

(d)

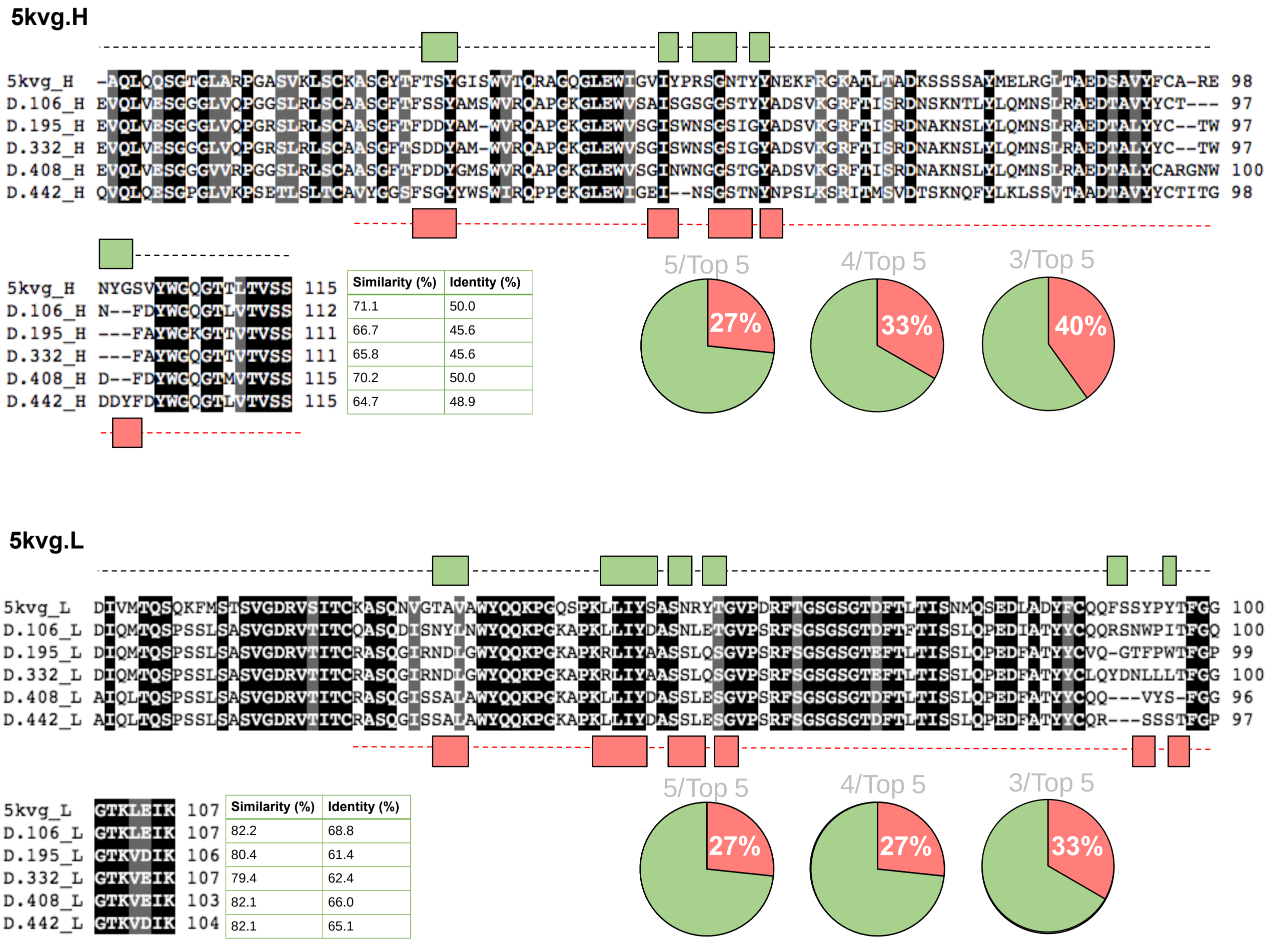

5kvg.H

	Similarity (%)	Identity (%)
5kvg_H		
D.106_H	71.1	50.0
D.195_H	66.7	45.6
D.332_H	65.8	45.6
D.408_H	70.2	50.0
D.442_H	64.7	48.9

5kvg.L

	Similarity (%)	Identity (%)
5kvg_L		
D.106_L	82.2	68.8
D.195_L	80.4	61.4
D.332_L	79.4	62.4
D.408_L	82.1	66.0
D.442_L	82.1	65.1

Figure S1 (**a**–**d**). Sequence alignments of the native heavy and light chain antibody sequences with the top five de novo designed sequences for (a) 5KVD, (b) 5KVE, (c) 5KVF, and (d) 5KVG respectively, have been represented. The green blocks represent native antibody residues which interact with the antigen epitope. The red blocks represent the antigen binding residues that are either seen in all the top five de novo designed variable domains or contribute significantly to the antigen binding affinity in at least one of the top five de novo designs. The three pie charts show the percentage of recovery of the native epitope binding interactions in (i) all five, (ii) any four, and (iii) any three of the top five de novo designed sequences.

Supplementary Figure 2:

(a)

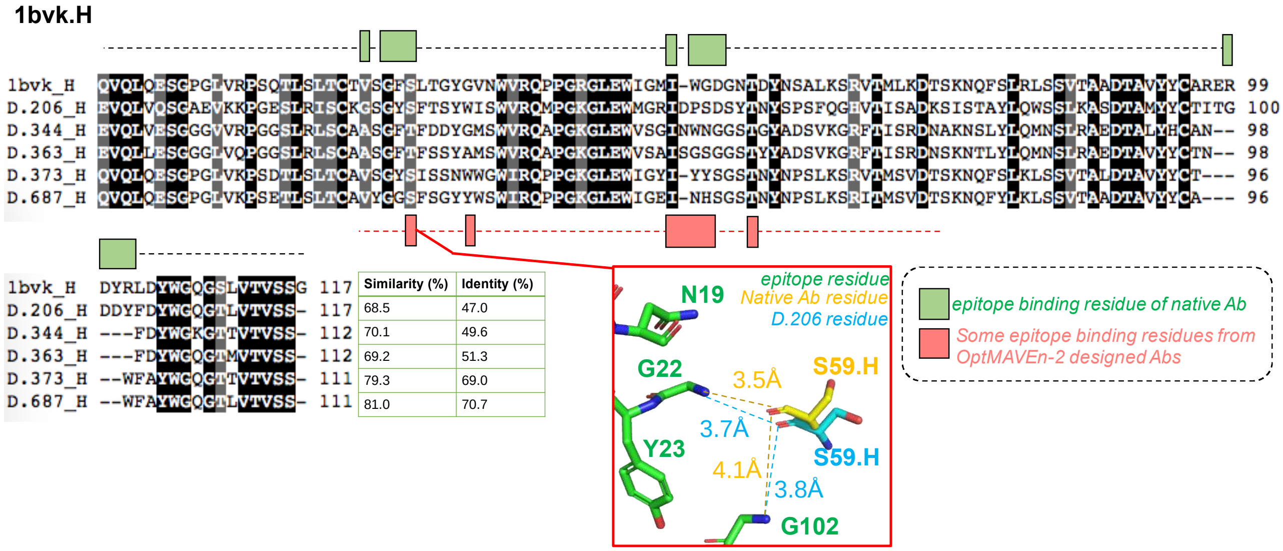

Similarity (%)	Identity (%)
68.5	47.0
70.1	49.6
69.2	51.3
79.3	69.0
81.0	70.7

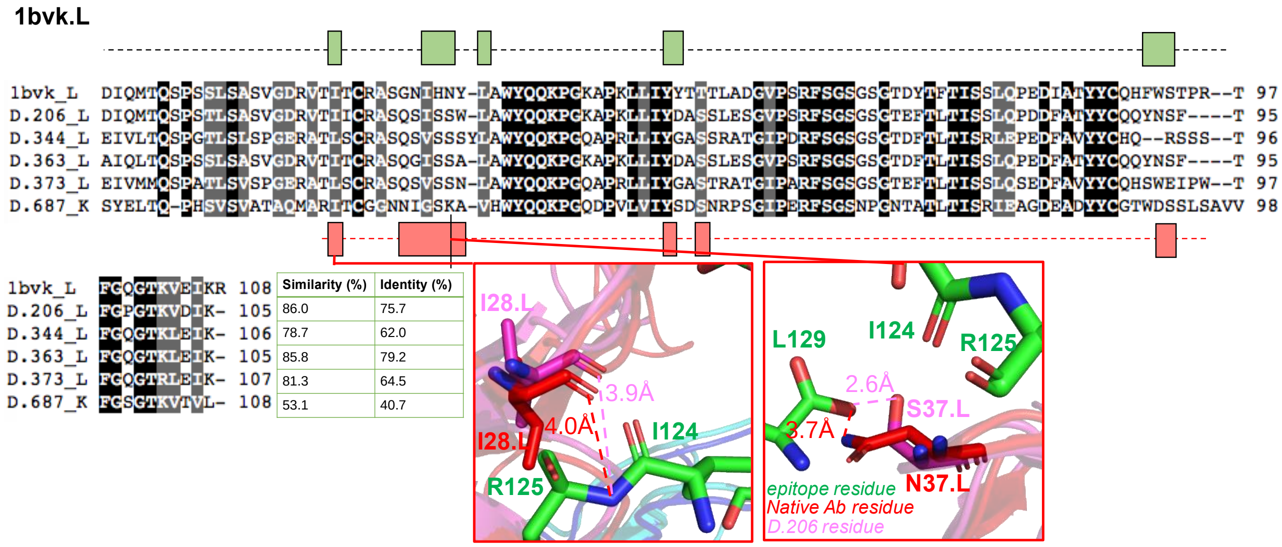

Similarity (%)	Identity (%)
86.0	75.7
78.7	62.0
85.8	79.2
81.3	64.5
53.1	40.7

(b)

4tsb.H

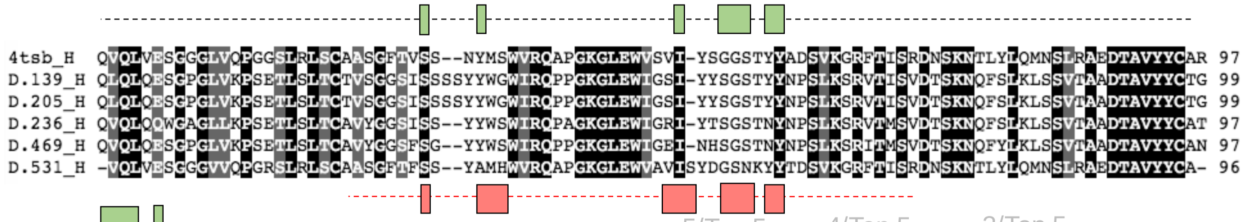

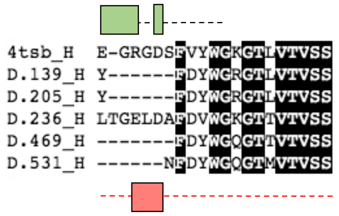

Similarity (%)	Identity (%)
68.9	56.3
68.9	56.3
69.5	55.1
68.4	56.4
85.5	80.3

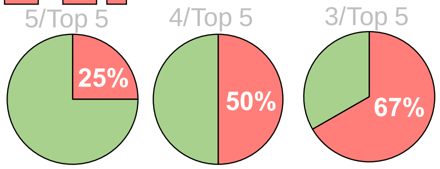

4tsb.L

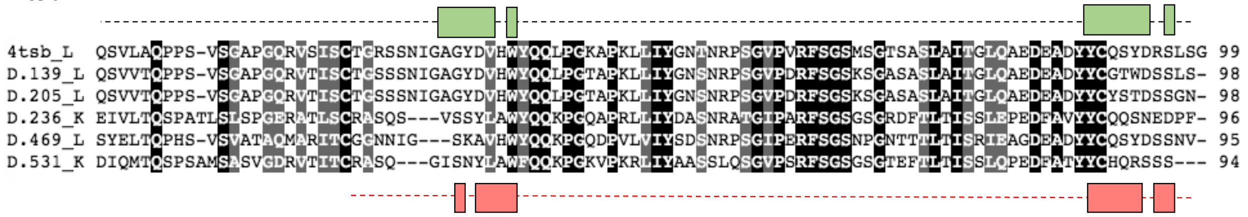

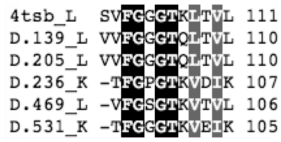

Similarity (%)	Identity (%)
91.0	85.6
88.3	84.7
65.1	46.8
70.4	57.4
62.5	49.0

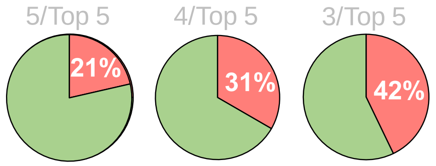

(c)

4pgj.H

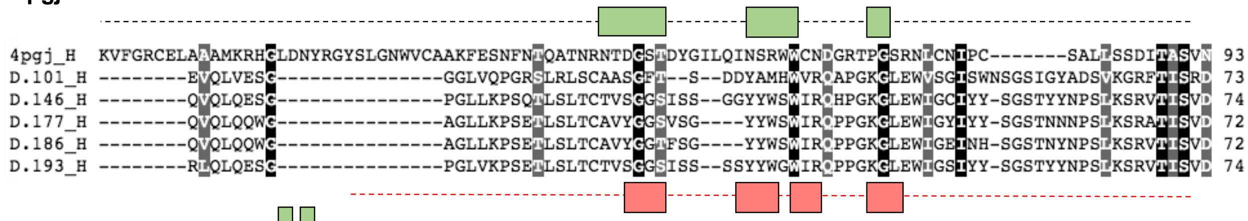

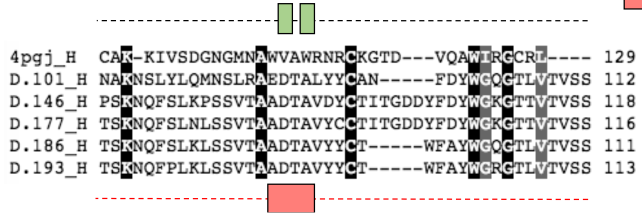

Similarity (%)	Identity (%)
44.5	25.0
44.4	31.1
45.5	27.3
43.2	27.3
37.2	25.6

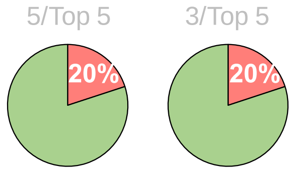

Figure S2 (a–c). Sequence alignments of the native heavy and light chain antibody sequences with the top five *de novo* designed sequences for (a) 1BVK, (b) 4TSB, and (c) 4PGJ have been represented. The insets in 2(a) show comparison of epitope interactions between the native antibody and the D.206 design. The green blocks represent native antibody residues which interact with the antigen epitope. The red blocks represent the epitope binding residues that are either seen in all the top five *de novo* designed variable domains or contribute significantly to the antigen binding affinity in at least one of the top five *de novo* designs.
